# Efficient Separation of C_2_H_6_/C_2_H_4_ and C_3_H_8_/C_2_H_6_/CH_4_ Light Hydrocarbons Using Robust Porous Polymer Networks for C_2_H_4_ and CH_4_ Purification

**DOI:** 10.1021/acsami.5c20065

**Published:** 2025-12-19

**Authors:** Kelechi Festus, Ankit Mondal, Fuan Guo, Sayan Maiti, Hengyu Lin, Vladimir Bakhmoutov, Hao Wang, Shengqian Ma, Lei Fang, Qingsheng Wang, Hong-Cai Zhou

**Affiliations:** † Department of Chemistry, 14736 Texas A&M University, College Station, Texas 77843, United States; ‡ Artie McFerrin Department of Chemical Engineering, 14736 Texas A&M University, College Station, Texas 77843, United States; § Hoffmann Institute of Advanced Materials, 47891 Shenzhen Polytechnic University, Shenzhen 518055, P. R. China; ∥ Department of Chemistry, 3404 University of North Texas, Denton, Texas 76203, United States

**Keywords:** selectivity, purification, light hydrocarbon, porous polymer networks, methane and ethylene

## Abstract

The efficient production of ultrahigh-purity C_2_H_4_ and CH_4_, which are vital feedstocks for industrial processes and alternative energy, is increasingly in demand but remains challenging. Cryogenic distillation has been widely utilized for this purpose; however, it imposes a substantial energy burden. Herein, we report four robust porous polymer networks (PPNs), namely, **PPN-35**, **PPN-45**, **PPN-55**, and **PPN-65,** for the purification of C_2_H_4_ from C_2_H_6_/C_2_H_4_ mixtures and CH_4_ from C_3_H_8_/C_2_H_6_/CH_4_ mixtures. **PPN-35**, **PPN-45**, **PPN-55**, and **PPN-65** exhibit IAST selectivities of 1.67, 1.43, 1.24, and 1.34 for C_2_H_6_/C_2_H_4_; 345.33, 199.16, 135.21, and 269.93 for C_2_H_6_/CH_4_; 20.17, 79.73, 31.77, and 15.98 for C_3_H_8_/C_2_H_6_; and 61907.55, 284358.59, 18175.83, and 19484.73 for C_3_H_8_/CH_4_, respectively. These values demonstrate that the materials are promising candidates to replace the widely used cryogenic distillation process. To the best of our knowledge, the C_2_H_6_/C_2_H_4_ selectivity of 1.67 achieved by **PPN-35** is the highest reported for any PPN, and the C_2_H_6_/CH_4_, C_3_H_8_/C_2_H_6_, and C_3_H_8_/CH_4_ selectivities surpassed all those of sorbents reported in the literature. Each of the four PPNs exhibits high adsorption capacities, and their practical usability was validated through breakthrough experiments, yielding >99% purity for both C_2_H_4_ and CH_4_. This work paves the way for exploring PPNs for light hydrocarbon separation.

## Graphical abstract

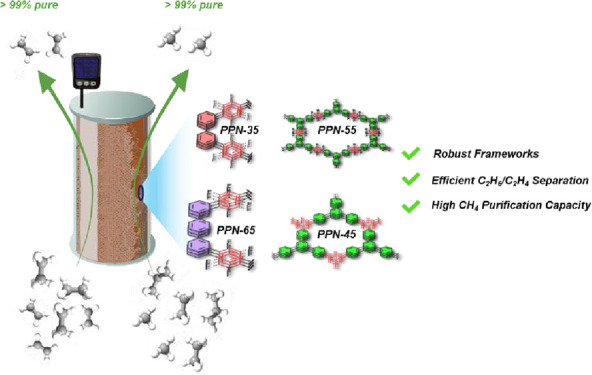

## 1 Introduction

The separation of light hydrocarbons has gained increasing attention because of their role in industrial processes and alternative energy. Despite their importance, separating and purifying them have been recognized as a challenging task with a tremendous energy penalty.^1^ Currently, cryogenic distillation technique is the widely used industrial method to separate them, but its significant energy consumption hinders the broader implementation of this technology.^2^ Although the use of cryogenic distillation is widely used in the industry, the development of an alternative method with minimal energy consumption has drawn significant attention.^3–5^ Evidently, the use of sorbents such as porous polymer networks (PPNs),^6^ metal–organic frameworks (MOFs),^3,7–12^ activated carbon,^13–15^ and zeolites^15–17^ has been investigated as alternatives. Sorbent-based separations rely on differences in kinetic diameter and molecular polarization, unlike cryogenic distillation, which exploits boiling point disparities and requires massive distillation towers. However, the fabrication of sorbents with precisely tuned pore sizes to selectively adsorb and purify the target gases remains a major challenge.^18^ Unlike MOFs, PPNs have not been widely studied or explored for this application despite their robustness, including their high thermal and chemical stability, pore tunability, and readily available monomers. This underscores the need to further explore PPNs as promising candidates for light hydrocarbon gas separation, as demonstrated by Wang et al.^6^

C_2_H_6_/C_2_H_4_ separation is crucial to afford ultrapure ethylene, a key industrial feedstock. C_2_H_4_ is especially important in the petrochemical industry, for the manufacture of polyethylene, which is often used to design various mechanical and electronic components and is commonly obtained on an industrial scale from natural gas.^19^ Although ethylene can be separated from ethane, this comes with a significantly high energy penalty, particularly because ethane is present only in small amounts, and its preferential removal from the C_2_H_6_/C_2_H_4_ mixture would significantly cut down process time and energy cost.^20–22^ This challenge has prompted the study of MOFs to achieve this goal, but the major drawback is that most of them are C_2_H_4_-selective instead of C_2_H_6_-selective. The consideration of PPNs as a promising candidate has not been thoroughly explored, despite their proven robustness in gas capture and storage (e.g., CO_2_).^23–26^ Ultimately, the development of a C_2_H_6_-selective sorbent instead of a C_2_H_4_-selective one would enable the single-step purification of a C_2_H_6_/C_2_H_4_ mixture and be energy-efficient.^27^ The few C_2_H_6_-selective PPNs reported to date for C_2_H_6_/C_2_H_4_ separation are PAN-P, PAN-NA, and PAN-AN, highlighting the need for the exploration and development of more novel and robust PPNs for these separations.^6^ To achieve this goal, the fabrication of PPNs with pore sizes engineered to fit the kinetic diameter of light hydrocarbons (for instance, C_2_H_6_ and C_2_H_4_, with diameters of 0.44 and 0.41 nm, respectively) is crucial to boost their molecular sieving capabilities.

Additionally, the global demand for affordable, cheap energy and the development of modern technology to mitigate global warming and climate change have become a global critical priority. Although CH_4_ is a potent greenhouse gas, it can serve as a low-cost energy source if harnessed before being released into the atmosphere.^28,29^ C_3_H_8_, C_2_H_6_, and CH_4_ are light hydrocarbon gases found in natural gas that must be separated to obtain high-purity CH_4_. Unlike these C_1_–C_3_ hydrocarbons, other heavier hydrocarbons, such as butane, can be liquified and removed easily, but the C_1_–C_3_ hydrocarbons are lighter and tedious to purify. Although cryogenic distillation remains the predominant method for this purpose, designing a more energy-efficient alternative is imperative. Sorbents such as MOFs and PPNs represent promising alternatives.^6,7^ Separating C_3_H_8_/C_2_H_6_/CH_4_ not only makes pure CH_4_ available but also yields C_3_H_8_, which can be used as a refrigerant or as a fuel source for industrial use, and C_2_H_6_, which serves as a feedstock for C_2_H_4_ production. The ability to synthesize PPNs with optimized pore sizes to achieve this goal, combined with their ultrahigh thermal and chemical stability due to hypercross-linking, makes them particularly promising candidates for this application.^30–32^ The availability of CH_4_ and C_3_H_8_ as low-cost sources of energy can significantly reduce the costs of industrial processes, manufacturing, and transportation. Moreover, capturing CH_4_ before release contributes to global warming mitigation.

PPNs are a class of sorbents fabricated from hypercross-linking of monomers to generate permanent porous structures. They are often categorized based on their pore diameter, which, according to the IUPAC classification, falls into one of three categories: microporous (<2 nm), mesoporous (2–50 nm), or macroporous (>50 nm).^33–35^ Owing to their unique properties, they have attracted attention for application in photocatalysis, light harvesting, chemical sensing, and gas capture and separation.^24,36–38^ Their pore size and morphology can also be tuned through rational reaction conditions and rigid monomer structure, which has broadened their application in various environmental and industrial processes.^39^ Several researchers have enhanced PPN performance through design, synthesis, and postsynthetic modification. However, their use in light hydrocarbon gas separation has not gained much attention, like that of MOFs and zeolites, which necessitates the need to develop new PPNs with light hydrocarbon purification potential. This highlight is further underscored by the robustness, cost-effectiveness, and stability of PPNs, which often exceed those of MOFs and zeolites.

Herein, we report a series of PPNs, namely, **PPN-35**, **PPN-45**, **PPN-55**, and **PPN-65**, for the separation of light hydrocarbon mixtures of C_2_H_6_/C_2_H_4_ and C_3_H_8_/C_2_H_6_/CH_4_ to produce high-purity C_2_H_4_ and CH_4_ for industrial applications and as a source of cheap energy. To the best of our knowledge, **PPN-35** has the highest C_2_H_6_/C_2_H_4_ selectivity of 1.67 for any PPN reported to date. Additionally, **PPN-35**, **PPN-45**, **PPN-55,** and **PPN-65** display record-high C_3_H_8_/CH_4_ (61907.55, 284358.59, 18175.83, and 19484.73), C_3_H_8_/C_2_H_6_ (20.17, 79.73, 31.77, and 15.98), and C_2_H_6_/CH_4_ (345.33, 199.16, 135.21, and 269.93) selectivity compared with values reported in the literature. These performances were verified through single-component gas adsorption, breakthrough experiments, Fourier-transform infrared spectroscopy (FTIR), and theoretical calculations of ideal adsorption solution theory (IAST) selectivity and isosteric heat of adsorption. The PPNs also demonstrated high thermal stability, which enables them to withstand harsh conditions during industrial applications, underscoring their robustness and efficiency.

## 2 Results and Discussion

A series of PPNs were synthesized via the Friedel–Crafts alkylation reaction from readily available monomers, producing thermally and chemically stable hypercross-linked networks, as shown in Figure 1a–d. The PPNs were synthesized as follows: **PPN-35** from biphenyl and 1,4-dibromotetrafluorobenzene (Figure 1a), **PPN-45** from 1,3,5-triphenylbenzene and cyanuric chloride (Figure 1b), **PPN-55** from 1,3,5-triphenylbenzene and 1,4-dibromotetrafluorobenzene (Figure 1c), and **PPN-65** from *p*-terphenyl and 1,4-dibromotetrafluorobenzene (Figure 1d). The irreversible polymerization reaction of these various monomers produced PPN moieties that are both very stable and amorphous. This was confirmed by thermogravimetric analysis (Figure S1), which indicates that all the sorbents exhibit high thermal stability, rendering them useful in harsh conditions and maintaining their structural integrity after multiple gas (C_3_H_8_, C_2_H_6_, C_2_H_4_, and CH_4_) uptake and separation cycles. The structure of the PPNs was further studied by using solid-state NMR to determine the bonding of the carbon atoms and the overall framework. Specifically, both ^13^C and ^19^F characterization were performed. The ^13^C ss-NMR spectrum shows peaks in the 110–160 ppm chemical shift range for **PPN-35**, **PPN-45**, **PPN-55**, and **PPN-65** (Figure S2a–d), which is indicative of the presence of aromatic carbons. In contrast, the upfield peaks show the presence of aliphatic carbons likely arising from solvents trapped within the pores of the PPNs. The ^19^F ss-NMR spectra showed no fluorine peaks for all the PPNs (Figure S3). The stacking of the spectra of all four PPNs shows a consistent absence of fluorine signals in the PPNs (Figure S4). The minor peaks observed originated from the ^19^F ss-NMR probe, indicating leaching of the fluorine atoms. This phenomenon has been previously reported for PPNs, as it has been observed that the carbon–fluorine bonds can be activated and cleaved under strong Lewis acid conditions.^40–43^ Powder X-ray diffraction (PXRD) confirmed the amorphous structure of the PPNs (Figure S5), attributed to the irreversibility of the polymerization process, unlike that of crystalline sorbents such as MOFs and covalent organic frameworks (COFs). This had a positive impact on the stability of the PPNs, as seen in Figure S1, and hence can withstand the stress, pressure, and temperature variations during adsorption and regeneration. FTIR spectra and transmission electron microscopy (TEM) micrographs of the sorbents further confirmed the successful synthesis of the sorbents (Figures S6 and S7). Due to the extensive cross-linking and interpenetration of the bonds during the polymerization process, direct visualization of individual pores of the sorbents was challenging. Despite this, the single-component gas adsorption (Figures S8–S11) and breakthrough experiments confirmed the presence of ultramicroporous (<0.7 nm) and microporous (<2 nm) pores in the sorbents. The presence of the ultramicropores in these sorbents (Figure 2a–d) is essential for the molecular sieving of the light hydrocarbon gas molecules and hence facilitates the enhanced selectivity and separation of C_2_H_6_/C_2_H_4_ and C_3_H_8_/C_2_H_6_/CH_4_ mixtures by **PPN-35**, **PPN-45**, **PPN-55**, and **PPN-65,** respectively.

**Figure 1:**
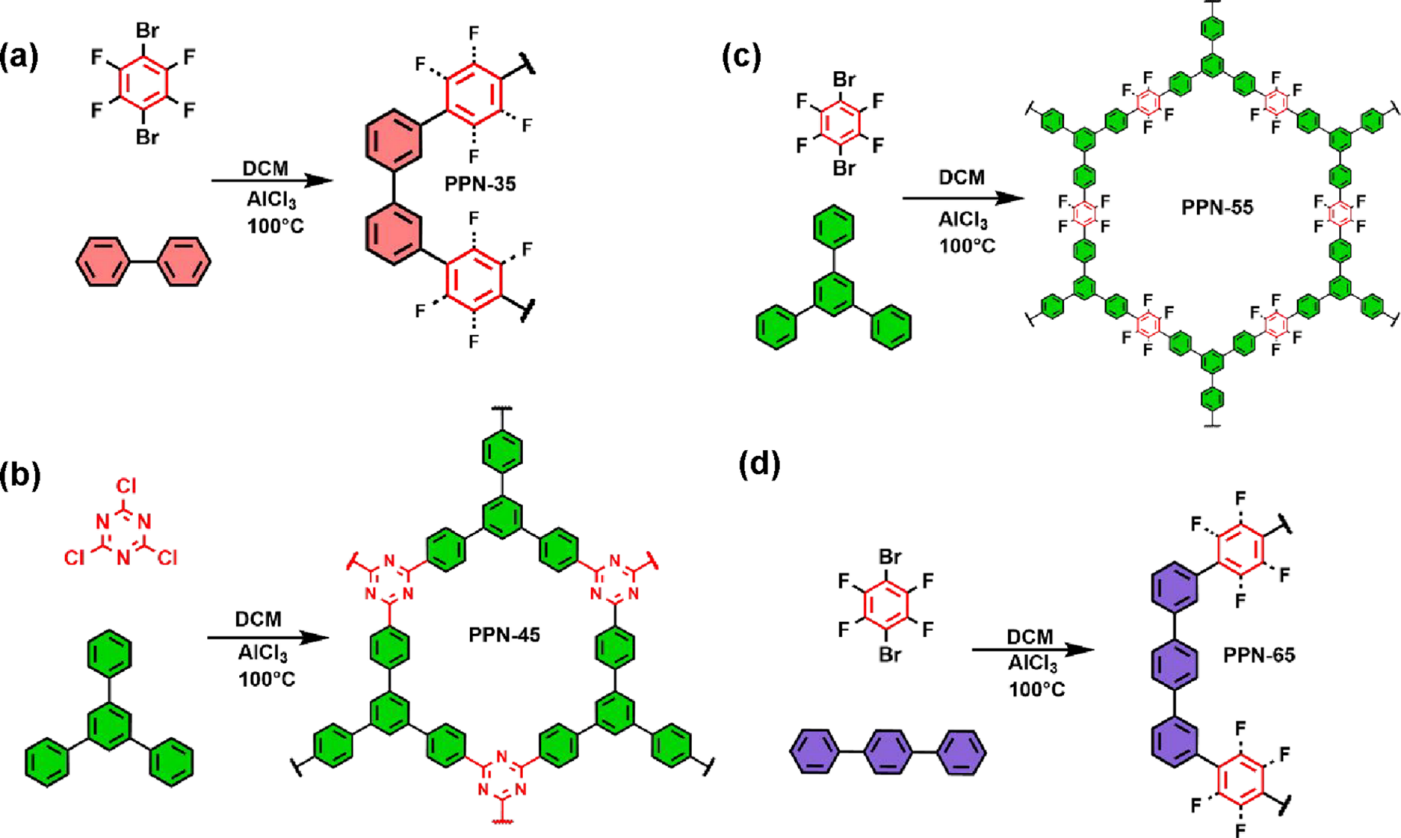
Synthetic scheme of ultramicroporous (a) **PPN-35**, (b) **PPN-45**, (c) **PPN-55**, and (d) **PPN-65**.

**Figure 2:**
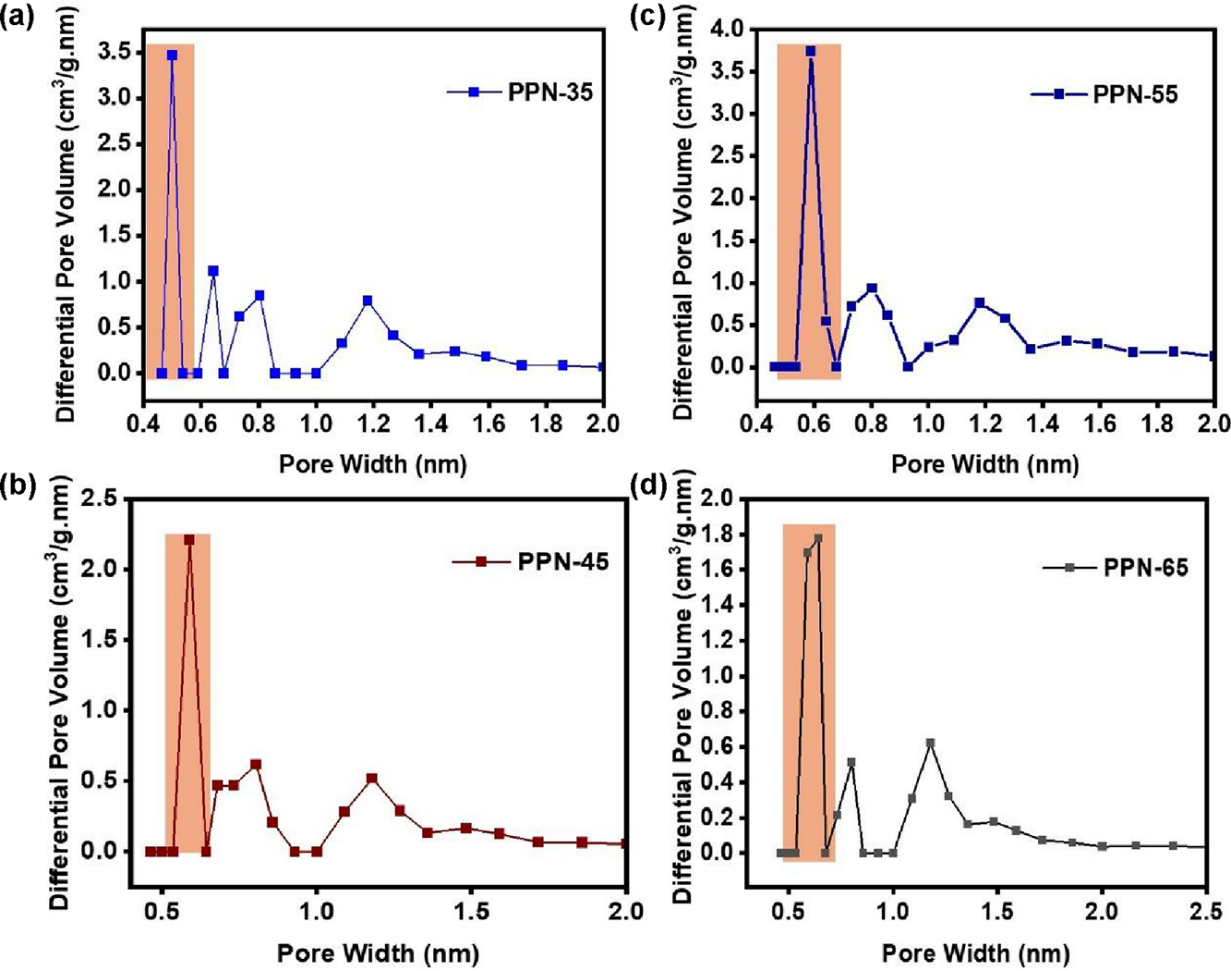
Pore size distribution of (a) **PPN-35**, (b) **PPN-45**, (c) **PPN-55**, and (d) **PPN-65** and their ultramicroporosity at 77 K and 1 bar.

To achieve the molecular sieving effect of the light hydrocarbon gases, ultramicroporosity contributes to the efficiency of the sorbents (**PPN-35**, **PPN-45**, **PPN-55**, and **PPN-65**). As shown in Figure S33, the kinetic diameters of these gases follow the order CH_4_ < C_2_H_4_ < C_2_H_6_ < C_3_H_8_. This trend guided the application of these sorbents for light hydrocarbon separation. The stability of the pores of the sorbents without collapsing contributed to the consistent performance observed after multiple adsorption–desorption cycles. The BET surface areas (m^2^/g) and pore size distribution of the sorbents were determined at 77 K and 1 bar from nitrogen (N_2_) sorption isotherms. **PPN-35**, **PPN-45**, **PPN-55**, and **PPN-65** have BET surface areas of 1637, 1254, 1104, and 1310 m^2^/g, respectively, reflecting their strong N_2_ adsorption capabilities (Figures S8–S11). Their pore widths were determined to be **PPN-35** (0.49 nm), **PPN-45** (0.58 nm), **PPN-55** (0.58 nm), and **PPN-65** (0.64 nm). This pore width implies that they all have ultramicroporous pores suitable for light hydrocarbon adsorption and separation, while PPN-35 exceeds due to its 0.49 nm.

Due to the presence of ultramicropores, the sorbents were further analyzed to validate their capability for light hydrocarbon gas adsorption (C_3_H_8_, C_2_H_6_, C_2_H_4_, and CH_4_). The single-component adsorption of C_3_H_8_, C_2_H_6_, C_2_H_4_, and CH_4_ was performed at 298 K and 1 bar (Figures S12–S16). It was observed that **PPN-35**, **PPN-45**, **PPN-55**, and **PPN-65** each had an uptake capacity of 5.06, 4.80, 4.27, and 3.57 mmol/g for C_3_H_8_; 3.52, 3.17, 3.30, and 2.80 mmol/g for C_2_H_6_; 3.18, 2.99, 3.14, and 2.62 mmol/g for C_2_H_4_; and 0.79, 0.69, 0.78, and 0.68 mmol/g for CH_4_, respectively, as summarized in Table S1. Notably, **PPN-35** showed the highest adsorption for each of the gases. The uptake pattern (C_3_H_8_ > C_2_H_6_ > C_2_H_4_ > CH_4_) observed for each of the sorbents is consistent with the molecular size order of the gas molecules, resulting in the preferential trapping of larger gas molecules by the ultramicropores. This consistent adsorption trend, governed by the kinetic diameter, molecular polarization, and molecular weight, established a baseline for evaluating their hydrocarbon separation capabilities (Table S2). IAST selectivity calculation was performed to determine their separation potential through extended Langmuir fitting on the single-component adsorption isotherms of each of the gases (C_3_H_8_, C_2_H_6_, C_2_H_4,_ and CH_4_) at 298 K and 1 bar (Figures S17–S24). For an equimolar (50/50) gas mixture, the IAST selectivities of **PPN-35**, **PPN-45**, **PPN-55**, and **PPN-65** are 1.67, 1.43, 1.24, and 1.34 for C_2_H_6_/C_2_H_4_, 345.33, 199.16, 135.21, and 269.93 for C_2_H_6_/CH_4_, 20.17, 79.73, 31.77, and 15.98 for C_3_H_8_/C_2_H_6_, and 61907.55, 284358.59, 18175.83, and 19484.73 for C_3_H_8_/CH_4_, respectively. **PPN-35** has the highest selectivity of C_2_H_6_/C_2_H_4_ reported for any PPN to the best of our knowledge, while all the four sorbents have higher selectivity than any of the state-of-the-art sorbents reported to date for CH_4_ purification, including ZUL-C2 ^44^ and Co-MOF.^45^ Although all four PPNs have a higher potential for CH_4_ purification than other reported sorbents, **PPN-35** stands out significantly because it has the highest IAST selectivity of 345.33 for C_2_H_6_/CH_4_.

The practical C_2_H_4_ separation performance of **PPN-35**, **PPN-45**, **PPN-55**, and **PPN-65** was studied through a dynamic breakthrough experiment with a C_2_H_6_/C_2_H_4_ (50/50, v/v) mixture to assess their suitability for producing industrial-grade C_2_H_4_. IAST selectivity for this binary gas mixture (Figure 3a) was used as a benchmark, as these PPNs demonstrated significant and robust gas separation capabilities compared to other reported sorbents (Figure 3b and Table S5). The dynamic breakthrough measurement performed in a fixed bed demonstrated that C_2_H_4_ breaks before C_2_H_6_ in all of the sorbents after a few minutes, with C_2_H_6_ having a longer retention time (Figure 3c–f). The purity of C_2_H_4_ was analyzed by a mass spectrometer attached to the breakthrough experiment setup to be >99% for each of the sorbents. As a result, they can be used to produce high-purity C_2_H_4_ from a C_2_H_6_/C_2_H_4_ mixture, which is essential for several industrial processes. The cyclability of the materials was screened to further elucidate their industrial relevance. This was determined through multiple C_2_H_6_/C_2_H_4_ breakthrough cycles under the same conditions without any loss or change in the selectivity or separation of C_2_H_4_ from C_2_H_6_ (Figure 4a–d).

**Figure 3:**
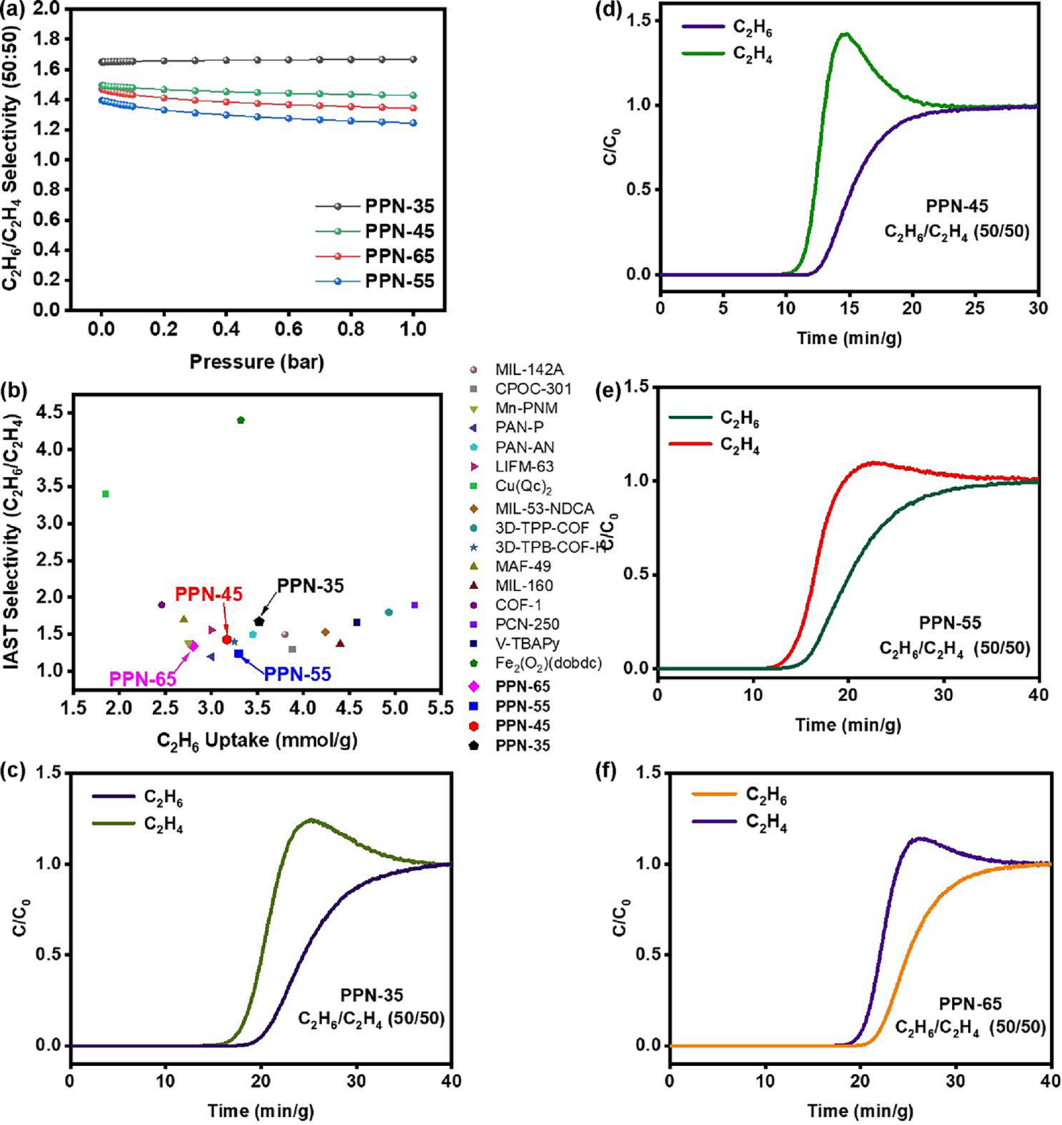
(a) IAST selectivity of C_2_H_6_/C_2_H_4_ for **PPN-35**, **PPN-45**, **PPN-55**, and **PPN-65** at 298 K and 1 bar. (b) Comparison of C_2_H_6_/C_2_H_4_ (50/50, v/v) IAST selectivity with other reported sorbents. Binary breakthrough curves for C_2_H_6_/C_2_H_4_ (50/50, v/v) of (c) **PPN-35**, (d) **PPN-45**, (e) **PPN-55**, and (f) **PPN-65** at 298 K and 1 bar.

**Figure 4:**
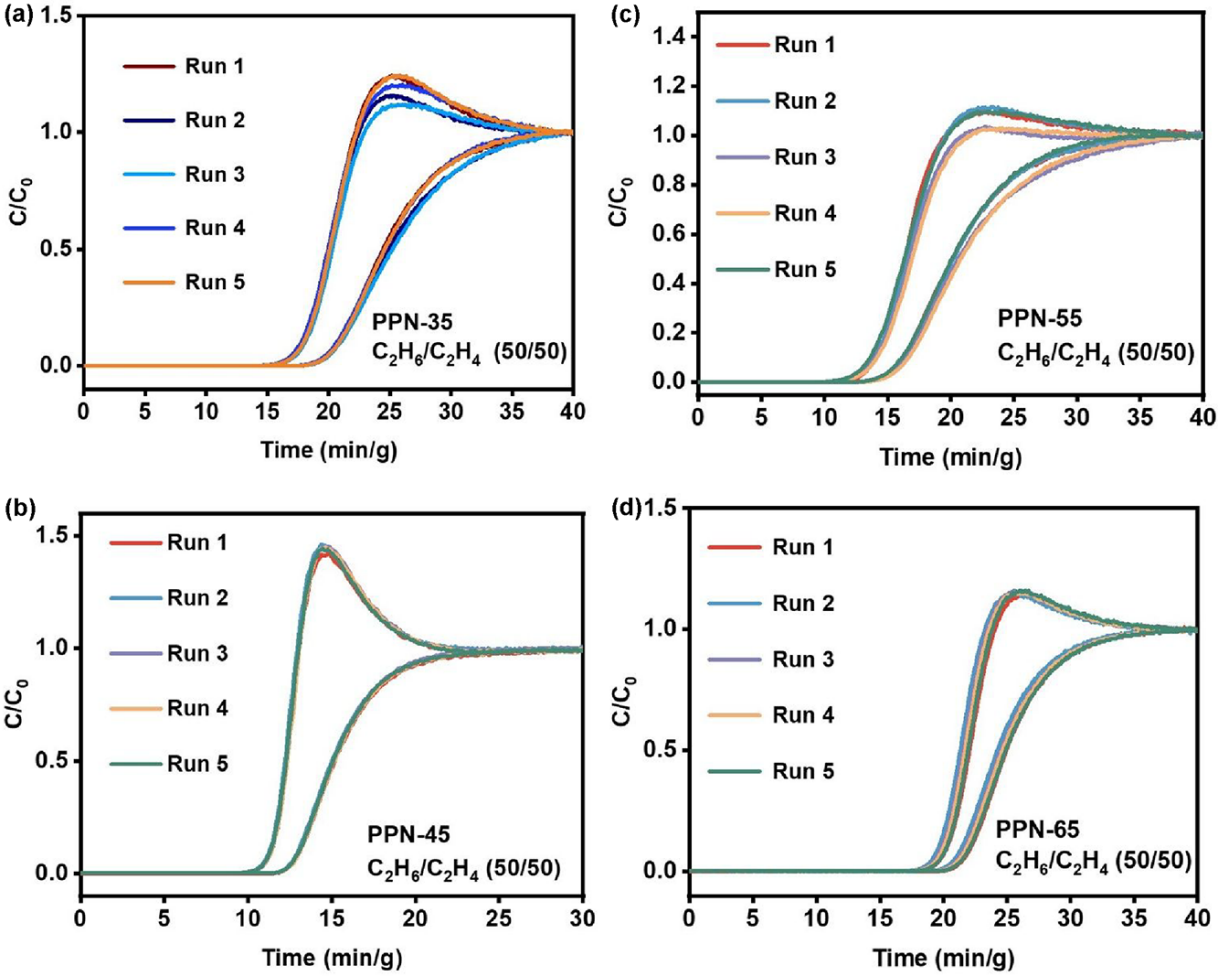
Binary mixed gas column breakthrough tests recycling of C_2_H_6_/C_2_H_4_ (50/50, v/v) separation for (a) **PPN-35**, (b) **PPN-45**, (c) **PPN-55**, and (d) **PPN-65** under ambient conditions.

The ability of the sorbents to retain their separation capability without any loss is traceable to their thermal stability (Figure S1) and amorphous structure (Figure S5), which makes their pores rigid against the stress of multiple passages of the C_2_H_6_ and C_2_H_4_ gas molecules in addition to the influence by the disparity in the kinetic diameter and polarizability of the gases (Figure S33 and Table S2). To further ascertain the behavior and compatibility of the gases with the PPNs, Langmuir–Freundlich fitting on the adsorption isotherms of C_3_H_8_, C_2_H_6_, C_2_H_4_, and CH_4_ at 273 K, 298 K, and 1 bar was used to calculate their isosteric heat of adsorption (*Q*_st_) in the pores of the sorbents (**PPN-35**, **PPN-45**, **PPN-55**, and **PPN-65**).^46^ C_3_H_8_ had the highest adsorption (mmol/g) and *Q*_st_ at zero and full loading (Tables S3 and S4), followed by C_2_H_6,_ among the other light hydrocarbon gases in the sorbents, because of their larger kinetic diameter and polarizability, which means that the selectivity observed is due to the molecular sieving effect of the PPN pores (Figure S33 and Table S2).

The importance of CH_4_ as a source of cheap energy and its role as a potent greenhouse gas cannot be overemphasized. Since CH_4_ in natural gas often contains C_2_H_6_ and C_3_H_8_ impurities requiring the use of a cryogenic distillation process for their purification, a C_3_H_8_/C_2_H_6_/CH_4_ (30/30/40, v/v/v) ternary mixed gas column breakthrough test was conducted on **PPN-35**, **PPN-45**, **PPN-55**, and **PPN-65**. This evaluation was motivated by their exceptionally high equimolar IAST selectivity in C_3_H_8_/CH_4_, C_2_H_6_/CH_4,_ and C_3_H_8_/C_2_H_6_ (Figure S34 and Table S6) at 298 K and 1 bar for CH_4_ purification (Figure 5a,b and Table S7). The IAST selectivity of the individual sorbents was compared to other best-performing sorbents (PPNs and MOFs) and outperformed them (Figure 5c,d and Table S7). This robustness was further reflected in the mixed gas (C_3_H_8_, C_2_H_6_, and CH_4_) column breakthrough curves of each of the sorbents (Figure 6a,d). Mass spectrometry analysis of the effluent confirmed that all four PPNs (**PPN-35**, **PPN-45, PPN-55**, and **PPN-65**) produced CH_4_ with >99% purity during the breakthrough experiments. This is because CH_4_ breaks first with significantly less breakthrough time compared to the other gases (CH_4_ < C_2_H_6_ and CH_4_ ≪ C_3_H_8_). The π-electron-rich aromatic rings in the PPNs also facilitated the selective adsorption of C_2_H_6_ and C_3_H_8_ because of their higher polarizability than CH_4_; this property was also up-tuned by the presence of ultramicroporous pores (<0.7 nm).^45^ Taken together, these results indicate that **PPN-35**, **PPN-45**, **PPN-55**, and **PPN-65** are highly promising for industrial-scale processing and purifying of CH_4_ from C_3_H_8_, C_2_H_6_, and CH_4_ mixtures. Essentially, this approach is effectively energy saving and an alternative to cryogenic distillation because the sorbents can be used for both C_2_H_6_/C_2_H_4_ or C_3_H_8_/C_2_H_6_/CH_4_ separations and purifications.

**Figure 5:**
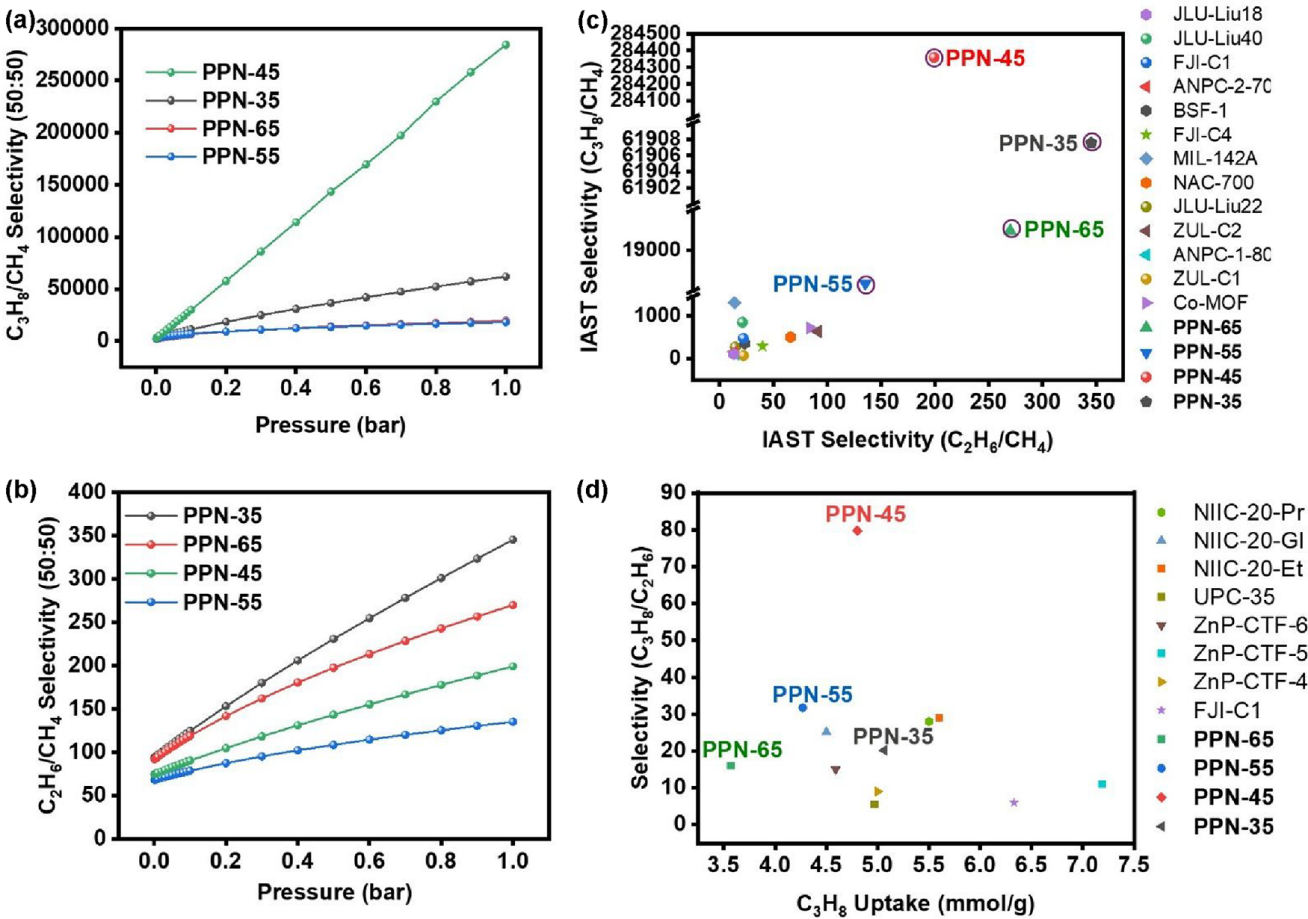
IAST selectivity of (a) C_3_H_8_/CH_4_ (50/50, v/v) and (b) C_2_H_6_/CH_4_ (50/50, v/v) at 298 K and 1 bar of **PPN-35**, **PPN-45**, **PPN-55**, and **PPN-65**. Comparison of the IAST selectivity performance of (c) C_3_H_8_/CH_4_ (50/50, v/v) and (d) C_2_H_6_/CH_4_ (50/50, v/v) compared to other reported sorbents.

**Figure 6:**
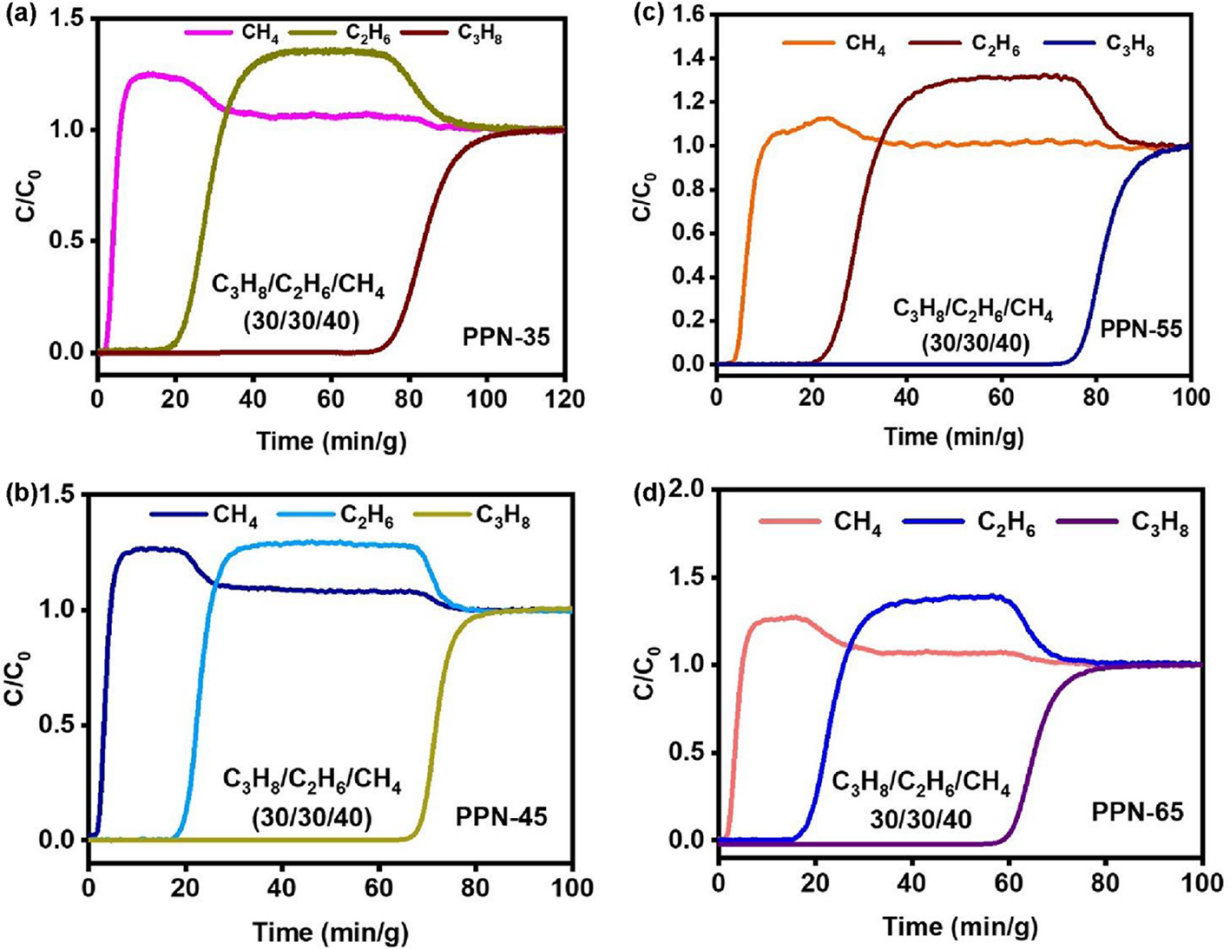
Ternary breakthrough curves of C_3_H_8_/C_2_H_6_/CH_4_ (30/30/40, v/v/v) gas mixture of (a) **PPN-35**, (b) **PPN-45**, (c) **PPN-55**, and (d) **PPN-65** at 298 K and 1 bar.

Ternary gas mixture separation and purification were further explored through multiple mixed gas breakthrough cycles of C_3_H_8_, C_2_H_6_, and CH_4_ on the PPNs **(PPN-35**, **PPN-45**, **PPN-55**, and **PPN-65)**. The breakthrough curves and retention times remained consistent, indicating that they are highly efficient for CH_4_ purification and can withstand multiple cycles. Typically, multiple breakthrough cycles introduce stress to the pores of sorbents and cause pore collapse, where pore rigidity is compromised. Still, these PPNs retained stability, which further makes them feasible for the commercial purification of CH_4_ in the industry (Figure 7a–d). The high IAST selectivity of each of the sorbents, the presence of ultramicropores, and molecular sieving effects were conclusively determined to be the driving force for these unique adsorption, selectivity, and separation properties. Thus, **PPN-35**, **PPN-45**, **PPN-55**, and **PPN-65** can be used industrially for the separation of C_2_H_6_/C_2_H_4_ to generate C_2_H_4_, an essential industrial monomer for manufacturing diverse polymer materials, since each sorbent generates C_2_H_4_ of >99% purity in one cycle. Furthermore, the ability of the sorbents to produce CH_4_ of >99% purity in a single cycle makes them suitable candidates as an energy-efficient alternative to cryogenic distillation. Even though CH_4_ is of utmost importance, the C_2_H_6_ and C_3_H_8_ generated from the separation and purification of the C_3_H_8_/C_2_H_6_/CH_4_ mixture are also useful since C_2_H_6_ can be used to produce C_2_H_4_ feedstock. At the same time, C_3_H_8_ serves as both a source of energy and a starting material for making C_3_H_6_ feedstock.^47–49^ In addition to CH_4_ as a source of cheap and clean energy, the C_3_H_8_ and C_2_H_6_ recovered from the mixed gas column breakthrough can hence still find practical applications, which makes it the first time, to the best of our knowledge, any PPN or a set of PPNs reported to possess the capability to separate both C_2_H_6_/C_2_H_4_ and C_3_H_8_/C_2_H_6_/CH_4_ mixtures to generate high-purity C_2_H_4_ and CH_4_, in addition to **PPN-35**, **PPN-45**, **PPN-55**, and **PPN-65** having IAST selectivities of 1.67, 1.43, 1.24, and 1.34 for C_2_H_6_/C_2_H_4_, 345.33, 199.16, 135.21, and 269.93 for C_2_H_6_/CH_4_, 20.17, 79.73, 31.77, and 15.98 for C_3_H_8_/C_2_H_6_, and 61907.55, 284358.59, 18175.83, and 19484.73 for C_3_H_8_/CH_4_ respectively. **PPN-35** has the highest IAST selectivity (1.67) reported for C_2_H_6_/C_2_H_4_ among any PPNs to date, while **PPN-35**, **PPN-45**, **PPN-55**, and **PPN-65** all exhibit significantly high IAST selectivity that surpasses any type of sorbent reported for CH_4_ purification from C_3_H_8_/C_2_H_6_/CH_4_ mixtures to date.

**Figure 7:**
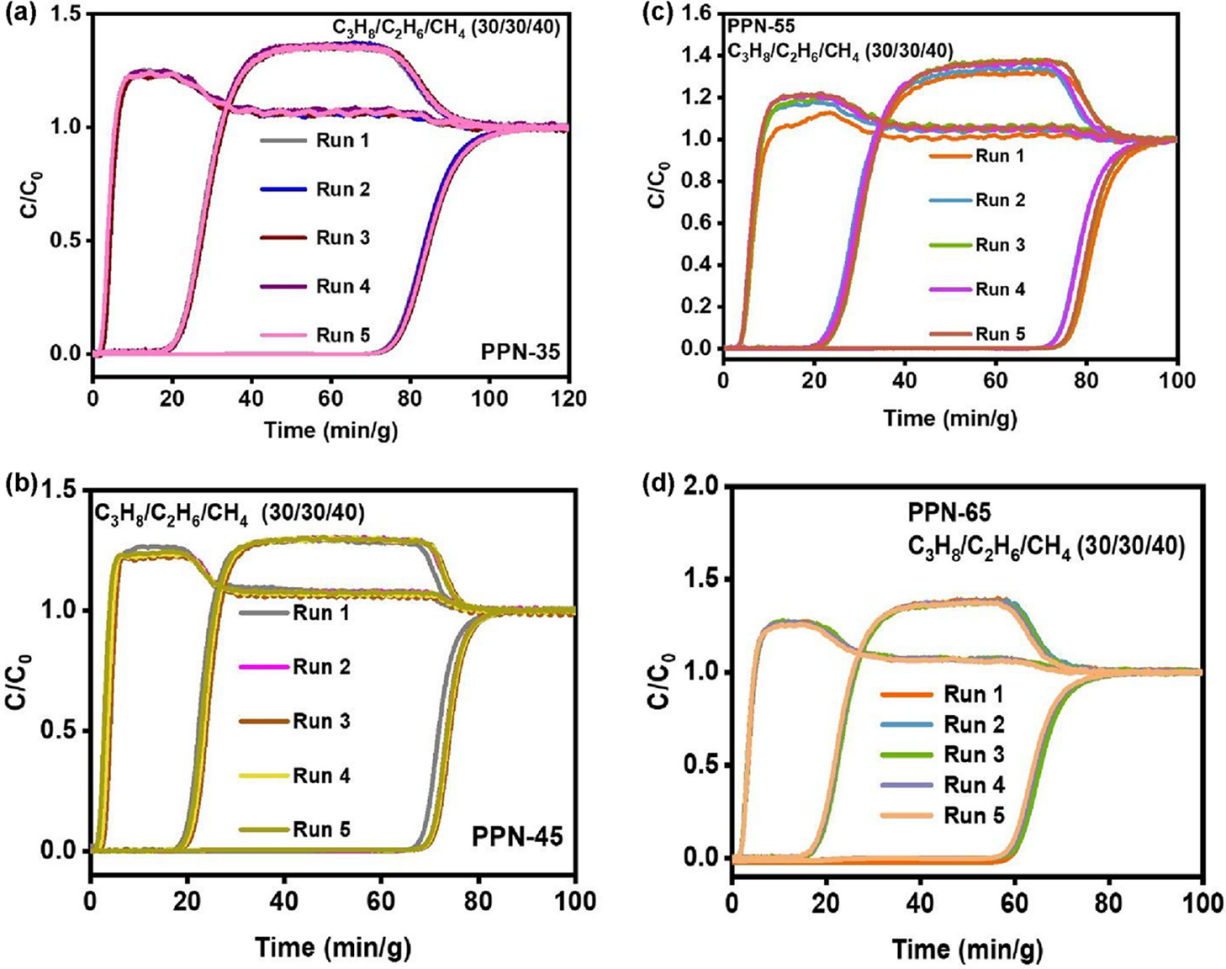
Ternary mixed gas column breakthrough tests recycling of a C_3_H_8_/C_2_H_6_/CH_4_ (30/30/40, v/v/v) gas mixture of (a) **PPN-35**, (b) **PPN-45**, (c) **PPN-55**, and (d) **PPN-65** under ambient conditions.

## 3 Conclusions

We have reported the synthesis of a group of PPNs for the separation of light hydrocarbon gases. Through controlled synthesis conditions and rational choice of rigid monomers, the PPNs (**PPN-35**, **PPN-45**, **PPN-55**, and **PPN-65**) were engineered to possess ultramicropores to preferentially adsorb and purify C_2_H_6_ and CH_4_ from C_2_H_6_/C_2_H_4_ and C_3_H_8_/C_2_H_6_/CH_4_ mixtures, respectively. Due to the ultramicroporosity, high IAST selectivity, and strong molecular sieving effect, **PPN-35** outperforms all other PPNs reported to date for C_2_H_6_/C_2_H_4_ selectivity. Due to the 0.49 nm pore width of **PPN-35**, it demonstrates a higher and robust molecular sieving capability by trapping larger molecules (CH_4_ < C_2_H_4_ < C_2_H_6_ < C_3_H_8_) more, which is consistent with their kinetic diameter, hence the observed superior selectivity. Additionally, all four sorbents surpass all known sorbents for C_3_H_8_/CH_4_, C_3_H_8_/C_2_H_6_, and C_2_H_6_/CH_4_ selectivity. The purity of the C_2_H_4_ and CH_4_ effluents from the mixtures (C_2_H_6_/C_2_H_4_ and C_3_H_8_/C_2_H_6_/CH_4_) was validated using a mass spectrometer in the mixed gas column breakthrough setup and was detected to be >99% pure, making it feasible for industrial applications. All four sorbents exhibited high thermal and chemical stability, retained their uptake and separation/purification capabilities after multiple gas mixture breakthrough cycles, and had an FTIR aromatic C=C ring stretching vibration at 1615 and 1495 cm^–1^. This performance will motivate the design and implementation of more robust PPNs to develop energy-efficient gas separation technologies. In addition, this work is expected to draw greater research attention to PPNs, a relatively underexplored class of materials for natural gas separation.

## 4 Experimental Section

### 4.1 Experiment Materials

All solvents and reagents used are commercially available and were used without further purification.

### 4.2 Synthesis of **PPN-35**

Biphenyl (0.373 g, 2.42 mmol), 1,4-dibromotetrafluorobenzene (0.744 g, 2.42 mmol), and anhydrous aluminum chloride (AlCl_3_) were transferred to a 50 mL round-bottom flask (RBM). Twenty ml of dichloromethane (DCM) was added to dissolve the mixture. The RBM containing the mixture was fitted with a condenser and heated at 100 °C for 24 h with moderate stirring. After completion, the reaction mixture was allowed to cool to room temperature, and the dark solid precipitate was collected by filtration. The solid was sequentially washed with water, tetrahydrofuran, dichloromethane, and methanol to remove the catalyst and unreacted monomers. It was then dried in a sand bath to obtain solvent-free **PPN-35.**

### 4.3 Synthesis of **PPN-45**

PPN-45 was synthesized following a reported procedure in the literature with modifications.^50,52^ Cyanuric chloride (0.470 g, 2.55 mmol) and anhydrous aluminum chloride (AlCl_3_) were dissolved in 10 mL of Chloroform in a 50 mL three-neck round-bottom flask (RBM). The mixture was stirred and heated for 30 min. 1,3,5-Triphenylbenzene (0.744 g, 2.43 mmol) was dissolved in 10 mL and added dropwise to the mixture. The three-neck RBM containing the mixture was fitted with a condenser and heated at 60 °C for 24 h with moderate stirring. After completion, the reaction mixture was cooled to room temperature, and the crude solid precipitate was obtained by filtration and sequentially washed with water, tetrahydrofuran, dichloromethane, and methanol to remove the catalyst and unreacted monomers. It was then dried in a sand bath to obtain a solvent-free **PPN-45.**

### 4.4 Synthesis of **PPN-55**

1,3,5-Triphenylbenzene (0.740 g, 2.42 mmol), 1,4-dibromotetrafluorobenzene (0.744 g, 2.42 mmol), and anhydrous aluminum chloride (AlCl_3_) were transferred to a 50 mL round-bottom flask (RBM). Twenty ml of dichloromethane (DCM) was added to dissolve the mixture. The RBM containing the mixture was fitted with a condenser and heated at 100 °C for 24 h with moderate stirring. After completion, the reaction mixture was cooled to room temperature, and the crude solid was collected by filtration and thoroughly washed with water, tetrahydrofuran, dichloromethane, and methanol sequentially to remove the catalyst and unreacted monomers to obtain a light brownish solid. It was then dried in a sand bath to obtain solvent-free **PPN-55.**

### 4.5 Synthesis of **PPN-65**

*p*-Terphenyl (0.556 g, 2.42 mmol), 1,4-dibromotetrafluorobenzene (0.744 g, 2.42 mmol), and anhydrous aluminum chloride (AlCl_3_) were transferred to a 50 mL round-bottom flask (RBM). Twenty ml of dichloromethane (DCM) was added to dissolve the mixture. The RBM containing the mixture was fitted with a condenser and heated at 100 °C for 24 h with moderate stirring. After completion, the reaction mixture was cooled to room temperature, and the dark solid precipitate was collected by filtration. The solid was sequentially washed with water, tetrahydrofuran, dichloromethane, and methanol to remove the catalyst and unreacted monomers. It was then dried in a sand bath to obtain solvent-free **PPN-65.**

### 4.6 Material Characterizations

A Bruker Avance-NEO solid-state NMR spectrometer (400 MHz for ^1^H nuclei) equipped with a standard two-channel 4 mm MAS probe head was used for the measurement of the ^19^F and ^13^C MAS NMR of the samples (**PPN-35**, **PPN-45**, **PPN-55**, and **PPN-65**). TMS and CCl_3_F were the external references for ^13^C and ^19^F nuclei, respectively. The ^19^F MAS NMR spectra were recorded with a single-pulse sequence (a pulse length of 3.4 μs), and 32 scans were applied at a spinning rate from 8 to 11 kHz using relaxation delays of 10 s. A spinning rate from 8 to 11 kHz was used to obtain the ^13^C MAS NMR spectra with a standard cross-polarization pulse sequence at a CP contact time of 1.2 ms (power of 77.2 W), 300–400 scans, and relaxation delays of 5 s. The standard *tppm15* pulse sequence has been used for high-power ^1^H decoupling. Transmission electron microscopy (TEM) images were obtained to determine the morphology of the various sorbents with a Titan Themis 300 S/TEM instrument (*RRID:SCR_022202*). Furthermore, the thermal stabilities were determined with a Mettler-Toledo TGA/DSC 1 at 5 °C/min from room temperature to 950 °C. Nicolet iS50 FTIR and a Molybdenum source Powder-ECO Bragg–Brentano: Theta/Theta: Lynxeye detector XT were used for Fourier-transform infrared spectroscopy (FTIR) and powder X-ray diffraction (PXRD) studies, respectively.

### 4.7 Gas Adsorption

To ascertain the porosity and surface area of **PPN-35**, **PPN-45**, **PPN-55**, and **PPN-65**, the samples were each placed in an ASAP 2020 Plus surface area and porosity analyzer sample tube and activated at 100 °C for 10 h at a heating rate of 10 °C/h and dosed with ultrahigh-purity (UHP) nitrogen at 77 K and 1 bar. The adsorption properties and capabilities of propane (C_3_H_8_), ethane (C_2_H_6_), ethylene (C_2_H_4_), and methane (CH_4_) uptake of each of the samples were measured at 298 K and 1 bar after activation at 120 °C and 600 min. To process the data, assuming a slit pore geometry, the data were processed with the Micromeritics MicroActive software.

### 4.8 Multicomponent Breakthrough Experiment

The binary and ternary gas breakthrough studies of the samples (**PPN-35, PPN-45**, **PPN-55**, and **PPN-65**) were measured with an auto mixed gas breakthrough apparatus (3P MIXSORB). 0.5 g portion of each sample was packed into a column (I.D. Six mm, volume 2 mL) and activated at 423 K for 2 h under a helium gas flow (10 mL/min) prior to the measurement. At a flow rate of 2 mL/min, the binary (C_2_H_6_/C_2_H_4_; 50/50 v/v) and ternary (C_3_H_8_/C_2_H_6_/CH_4_; 30/30/40, v/v/v) gas mixtures and helium gas at a flow rate of 6 mL/min were switched to pass through the adsorption bed, and the outlet gas was analyzed with a mass spectrometer (MKS circus 3). After the adsorption reached dynamic equilibrium, the column was purged with He (10 mL/min) at 423 K for 2 h for regeneration.

### 4.9 Isosteric Heat of Adsorption

At 298 K and 1 bar, the isosteric heat of adsorption was calculated from the single-component adsorption isotherms of each of the light hydrocarbon gases (C_3_H_8_, C_2_H_6_, C_2_H_4_, and CH_4_) to determine the interaction of the various gases and each sorbent. The isotherms were fitted with a Langmuir–Freundlich equation^46^ shown below:

$$y=\frac{a\cdot b\cdot p^{c}}{1+b\cdot p^{c}}$$ 1

The description of the above equation is as follows. *y* is the adsorbed quantity (mmol/g). *a* (mmol/g) and *b* (1/kPa^*c*^) are the adsorption capacity and the adsorbate and adsorbent affinity strength, respectively. *c* stands for the dimensionless deviation from the ideal homogeneous surface. *p* is the pressure in kPa. The Clausius–Clapeyron equation below was used to determine the enthalpy of adsorption of the various gases on the sorbent.

$$\frac{\partial (\ln\;P)}{\partial (1/T)}=-\frac{Q_{\mathrm{st}}}{R}$$ 2

*P*, *T*, *R*, and *Q*_st_ stand for gas pressure, adsorption temperature, universal gas constant, and enthalpy of adsorption, respectively.

### 4.10 IAST Selectivity

The extended Langmuir equation was used to fit the adsorption isotherms of C_3_H_8_, C_2_H_6_, C_2_H_4_, and CH_4_ to determine the ideal adsorption solution theory (IAST) selectivity of C_2_H_6_/C_2_H_4_, C_3_H_8_/CH_4_, C_3_H_8_/C_2_H_6_, and C_2_H_6_/CH_4_ in a 50/50 mixture ratio at 298 K and 1 bar.

$$\begin{array}{c} y=\frac{a\cdot b\cdot p^{1-c}}{1+b\cdot p^{1-c}} \end{array}$$ 3

The description of the above equation is as follows. *y* is the adsorbed quantity (mmol/g). *a* (mmol/g) and *b* (1/kPa^*c*^) are the adsorption capacity and the adsorbate and adsorbent affinity strength, respectively. *c* stands for the dimensionless deviation from the ideal homogeneous surface. *p* is the pressure in kPa. The Myers and Prausnitz^51^ equation below was used to determine the selectivity of the various gases on the sorbent.

$$\begin{array}{c} S_{\mathrm{ads}}=\frac{q_{1}/q_{2}}{p_{1}/p_{2}} \end{array}$$ 4

The parameters of the equation simplify as *S*_ad_ being the adsorption selectivity and *q*_1_ and *q*_2_ representing the mole fractions of the gases whose adsorptions are compared and calculated at specific ratios.

## Supplementary Material



## Notes

TGA curve, ss-NMR, PXRD, FTIR, TEM, gas adsorption, IAST selectivity fitting and plots, *Q*_st_ fitting and plots, light hydrocarbon size comparison, summary of light hydrocarbon adsorption, physical properties of light hydrocarbons, summary of IAST selectivity and Qst of light hydrocarbons, and performance comparison with other sorbents (PDF)
