# Supplementary material for: Efficient Separation of C2H6/C2H4 and C3H8/C2H6/CH4 Light Hydrocarbons Using Robust Porous Polymer Networks for C2H4 and CH4 Purification
Source: ACS Appl Mater Interfaces. 2025 Dec 19;18(1):2444–54. doi: 10.1021/acsami.5c20065 (PMC12781049; doi:10.1021/acsami.5c20065)
Supplement: Supplementary file 1 [file am5c20065_si_001.pdf]

Supporting Information

Efficient Separation of C_2H_6/C_2H_4 and $C_3H_8/C_2H_6/CH_4$ Light Hydrocarbons Using Robust Porous Polymer Networks for C_2H_4 and CH_4 Purification

Kelechi Festus[1,2], Ankit Mondal[1], Fuan Guo[3], Sayan Maiti[4], Hengyu Lin[1], Vladimir Bakhmoutov[1], Hao Wang[3], Shengqian Ma[4], Lei Fang[1], Qingsheng Wang[2]* & Hong-Cai Zhou[1]*

[1]Department of Chemistry, Texas A&M University, College Station, TX 77843, USA

[2]Artie McFerrin Department of Chemical Engineering, Texas A&M University, College Station, TX 77843, USA

[3]Hoffmann Institute of Advanced Materials, Shenzhen Polytechnic University, Shenzhen 518055, P. R. China

[4]Department of Chemistry, University of North Texas, Denton, TX 76203, USA

*Corresponding Authors

Q. Wang (qwang@tamu.edu); H.-C. Zhou (zhou@chem.tamu.edu)

Supporting Figures and Tables

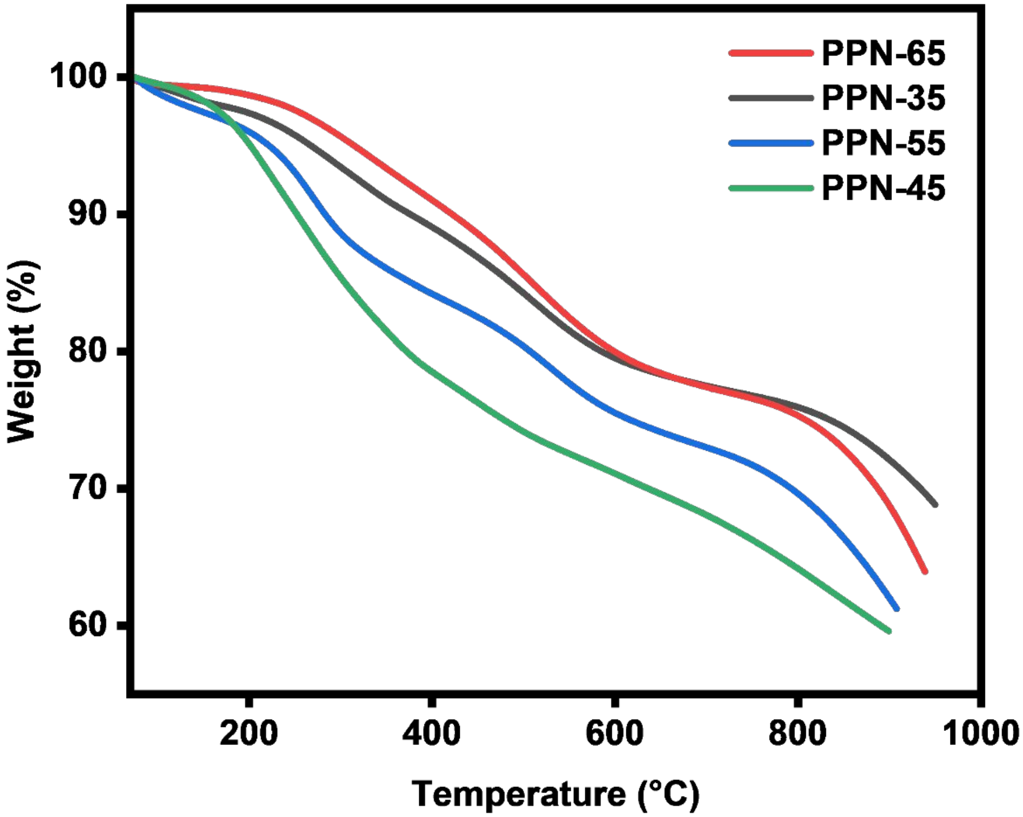

Figure S1. Thermogravimetric Analysis (TGA) curves of **PPN-35**, **PPN-45**, **PPN-55**, and **PPN-65**.

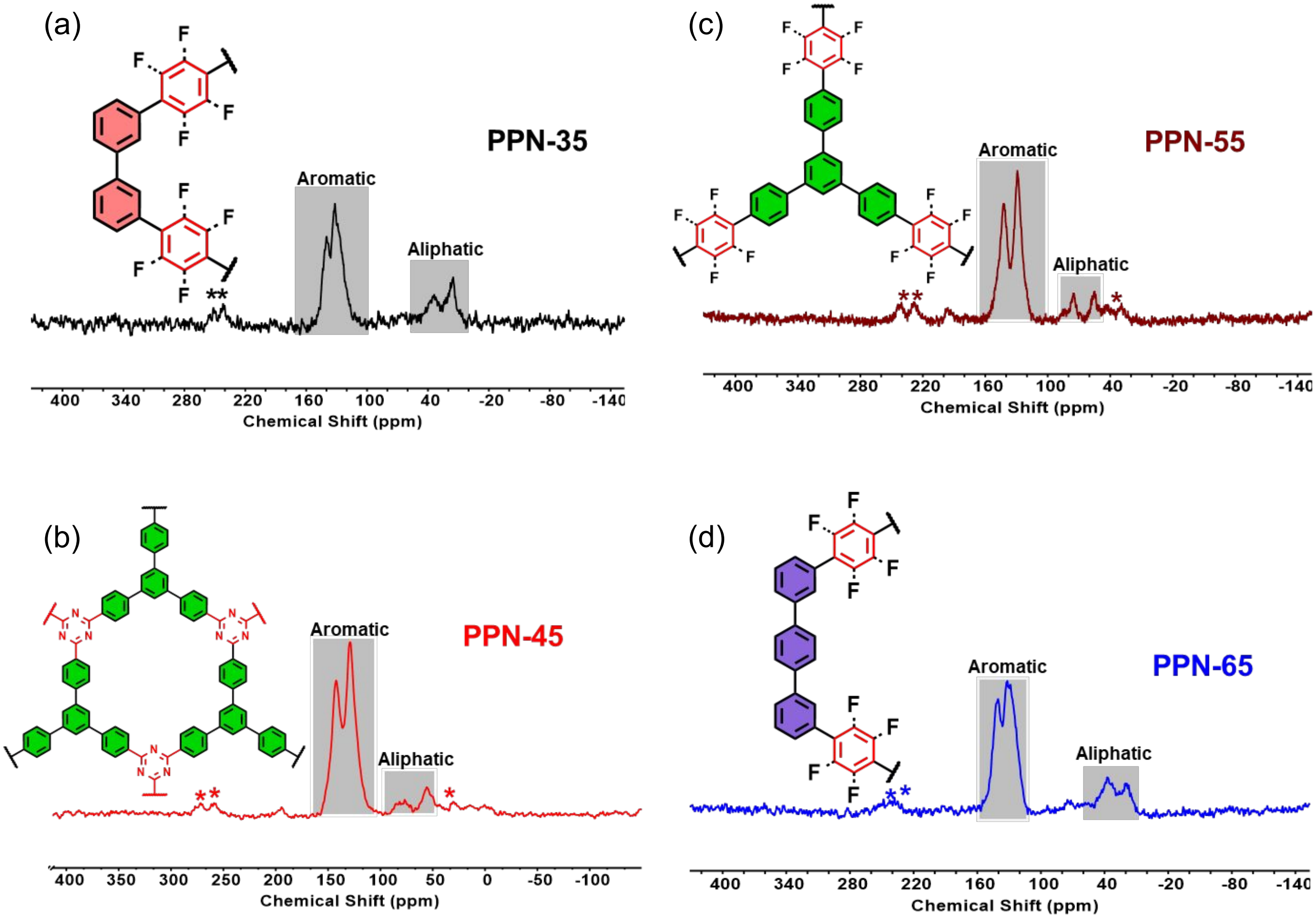

Figure S2. Solid-state ^{13}C CP/MAS NMR spectra of porous polymer networks (PPNs): (a) **PPN-35**, (b) **PPN-45**, (c) **PPN-55**, and (d) **PPN-65**, highlighting aromatic (100–160 ppm) and aliphatic (0–70 ppm) regions.

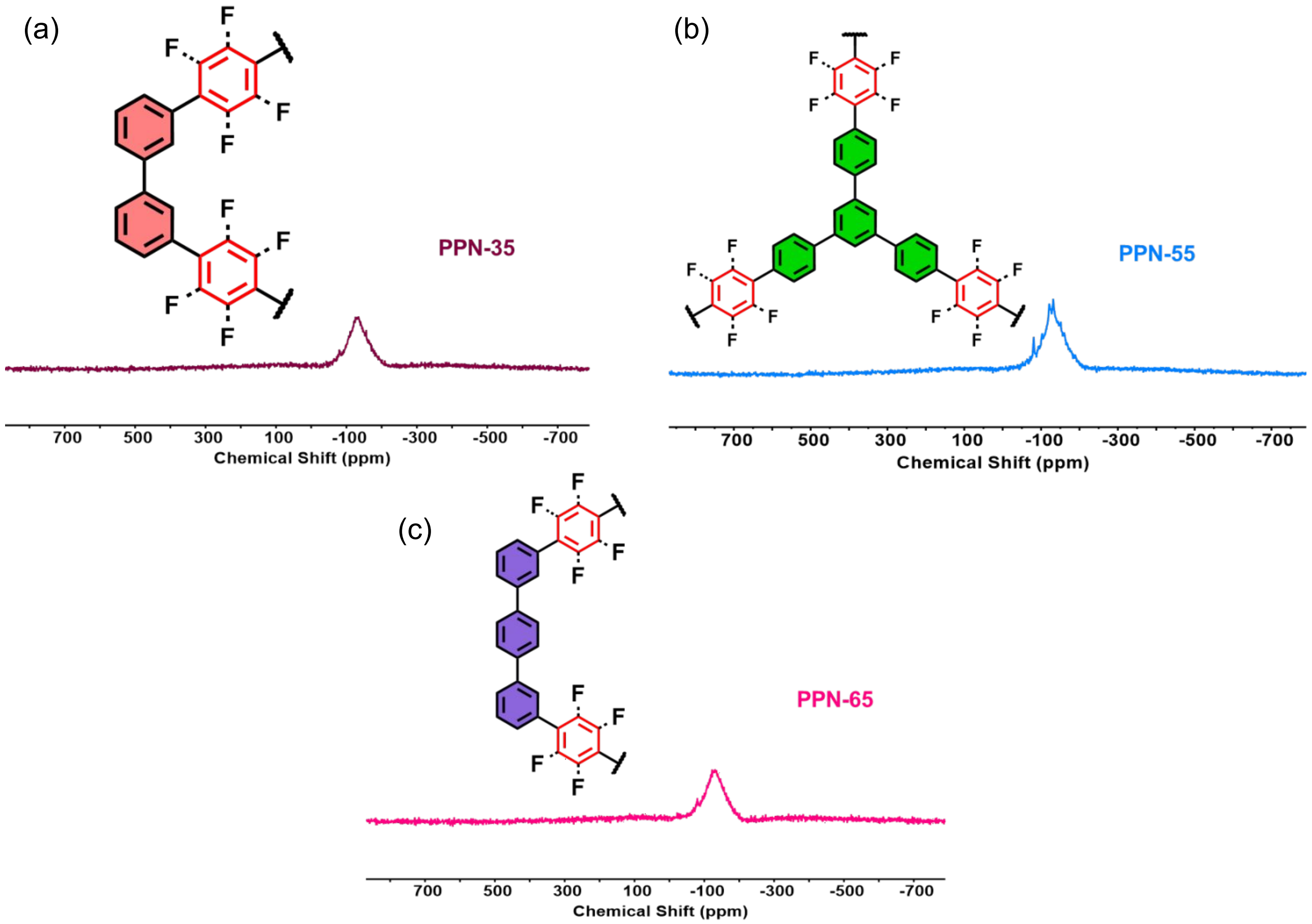

Figure S3. ^{19}F solid-state NMR of the PPNs: (a) **PPN-35**, (b) **PPN-55**, (c) **PPN-65**. This highlights the absence of fluorine atoms in the network. The peak at around -100 ppm is from the ss-NMR probe.

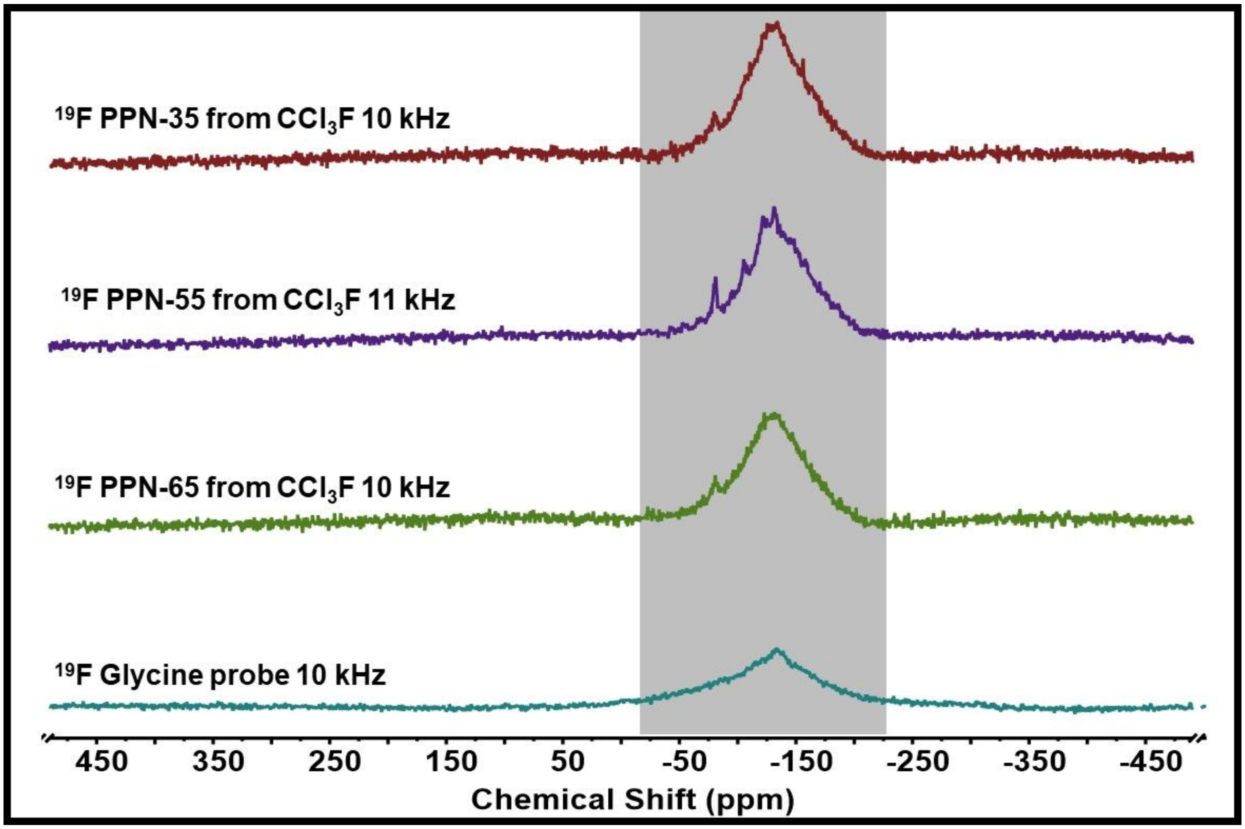

Figure S4. Comparison of the ^{19}F solid-state NMR spectra of four PPNs (**PPN-35**, **PPN-45**, **PPN-55**, and **PPN-65**), which validates the defluorination of the fluorine atoms in the PPNs.

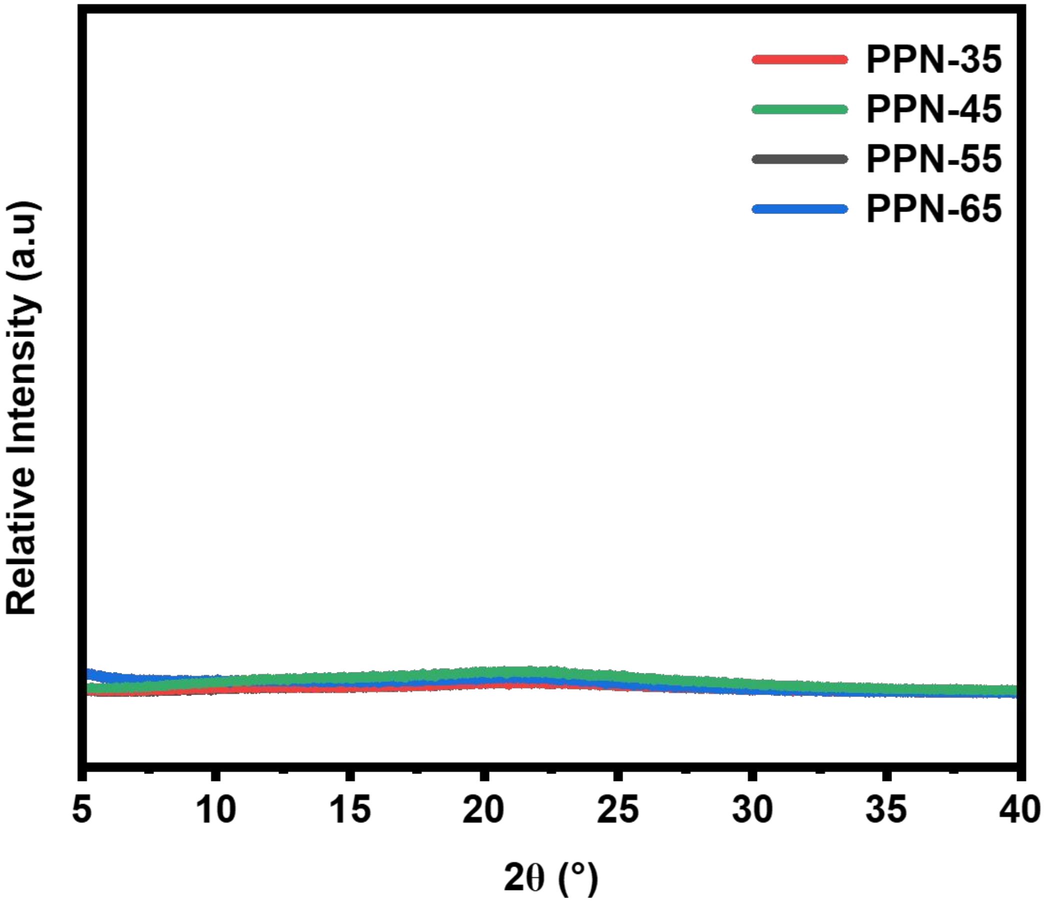

Figure S5. Powder X-ray diffraction (PXRD) patterns of **PPN-35**, **PPN-45**, **PPN-55**, and **PPN-65**, confirm their amorphous nature.

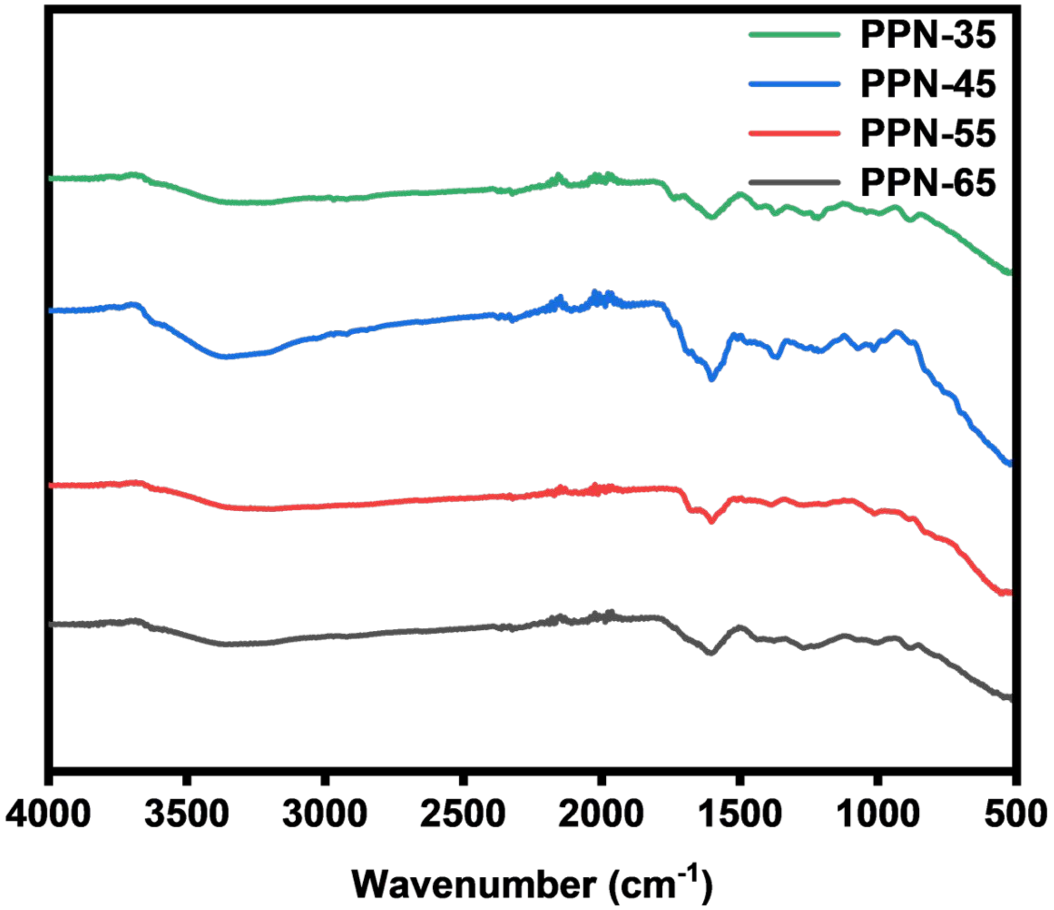

Figure S6. FTIR Spectra of the four porous polymer networks (**PPN-35**, **PPN-45**, **PPN-55**, and **PPN-65**).

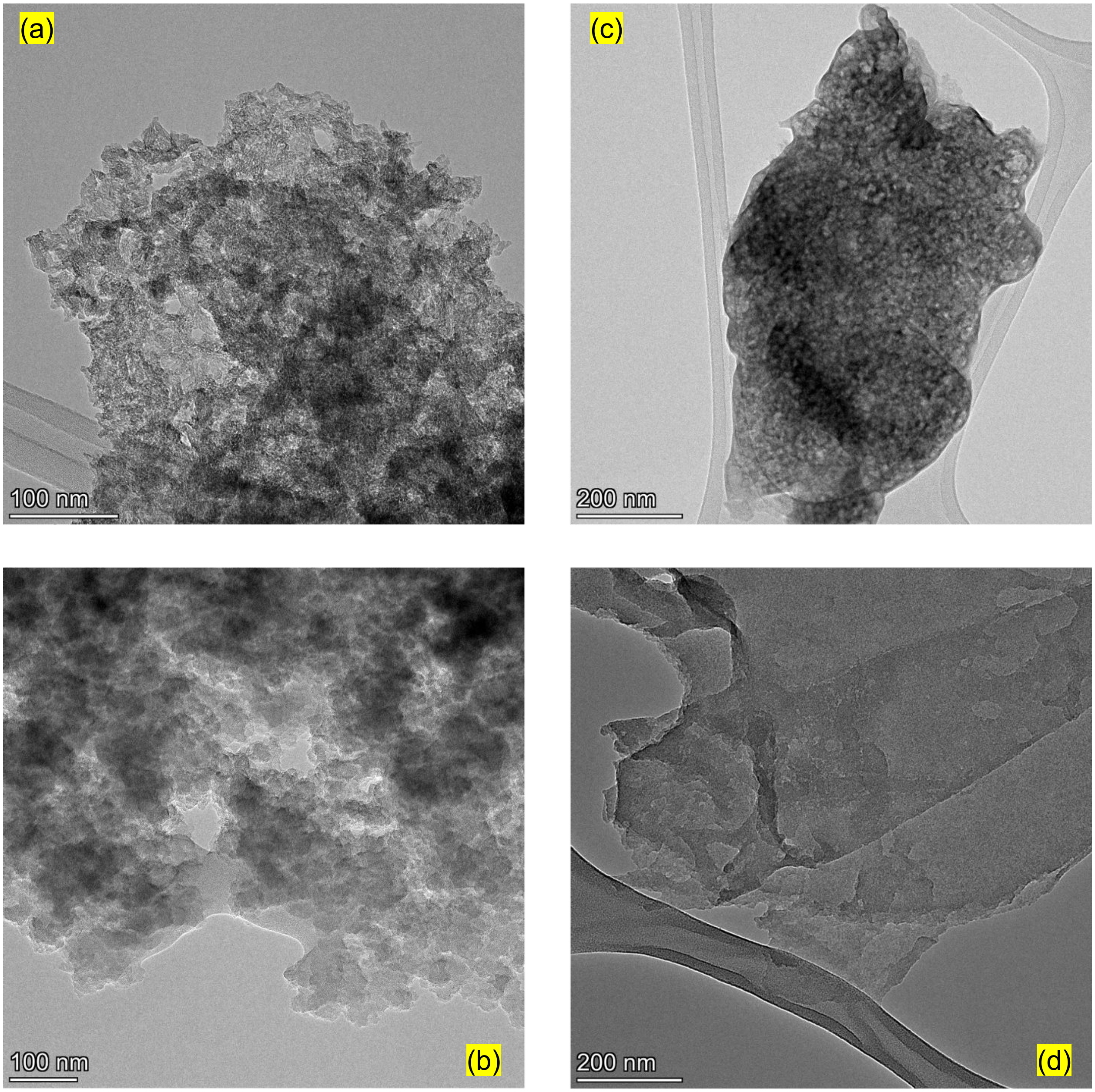

Figure S7. Transmission Electron Microscopy (TEM) cross-sectional images of the porous polymer networks: (a) **PPN-35**, (b) **PPN-45**, (c) **PPN-55**, and (d) **PPN-65**. Scale bars: 100 nm for (a) and (b); 200 nm for (c) and (d).

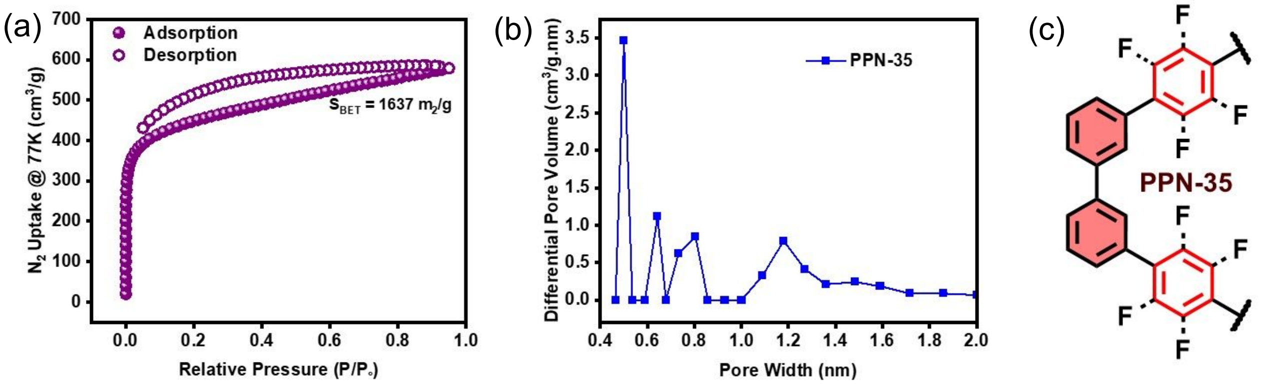

Figure S8. (a) Nitrogen sorption isotherm and BET surface area of **PPN-35** at 77 K and 1 bar. (b) Pore size distribution of **PPN-35** at 77 K and 1 bar. (c) The chemical scaffold of **PPN-35**.

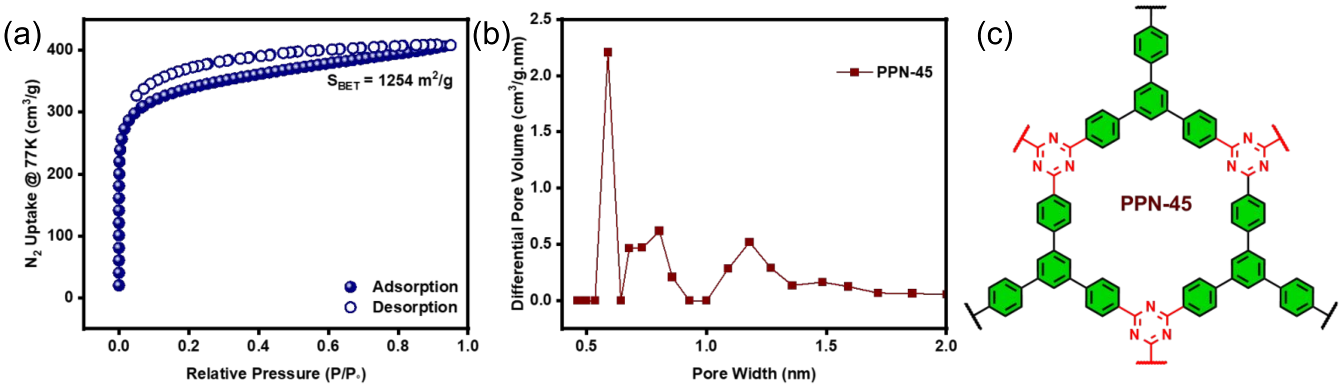

Figure S9. (a) Nitrogen sorption isotherm and BET surface area of **PPN-45** at 77 K and 1 bar. (b) Pore size distribution of **PPN-45** at 77 K and 1 bar. (c) The chemical scaffold of **PPN-45**.

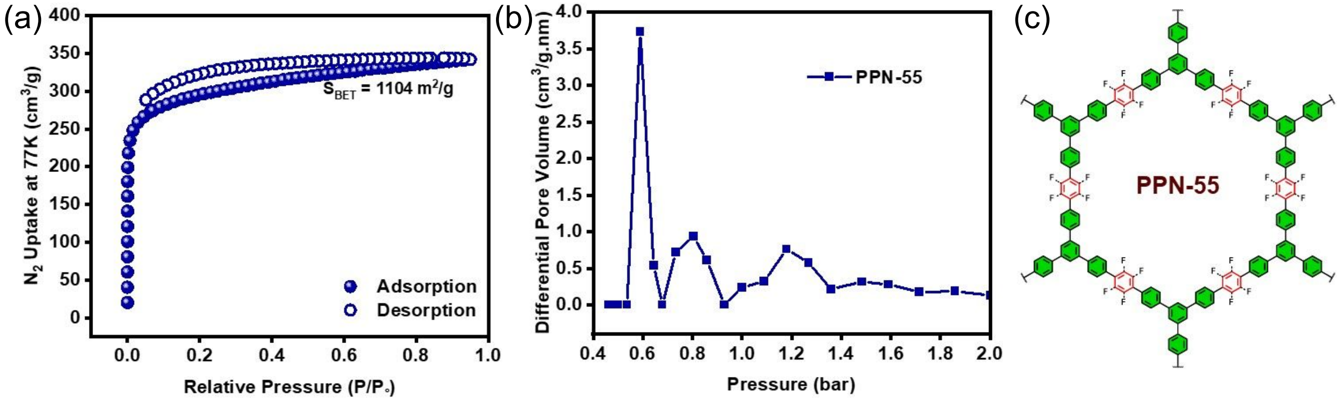

Figure S10. (a) Nitrogen sorption isotherm and BET surface area of **PPN-55** at 77 K and 1 bar. (b) Pore size distribution of **PPN-55** at 77 K and 1 bar. (c) The chemical scaffold of **PPN-55**.

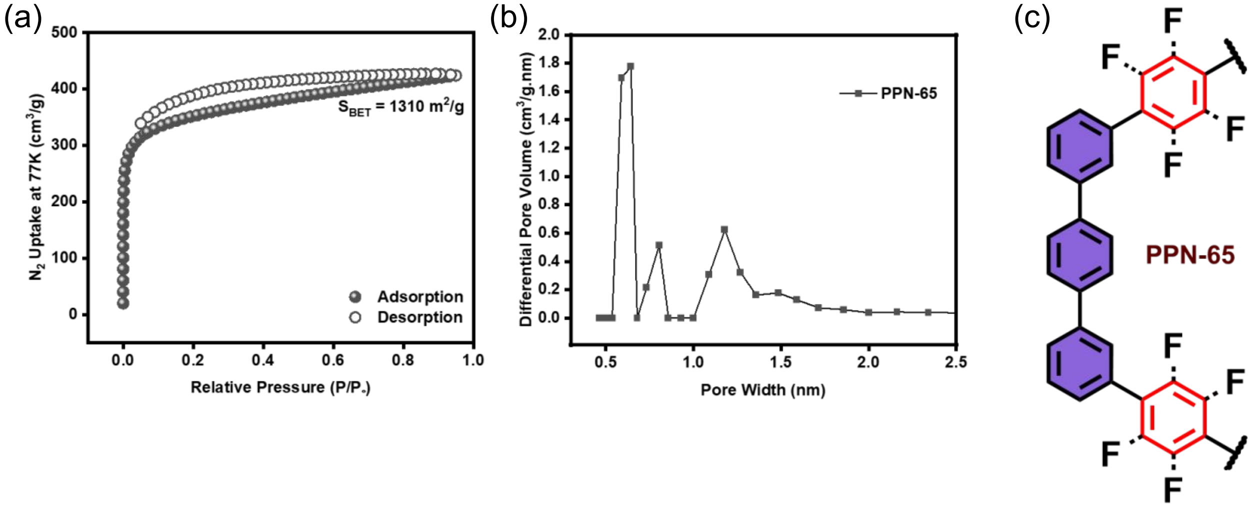

Figure S11. (a) Nitrogen sorption isotherm and BET surface area of **PPN-65** at 77 K and 1 bar. (b) Pore size distribution of **PPN-65** at 77 K and 1 bar. (c) The chemical scaffold **PPN-65**.

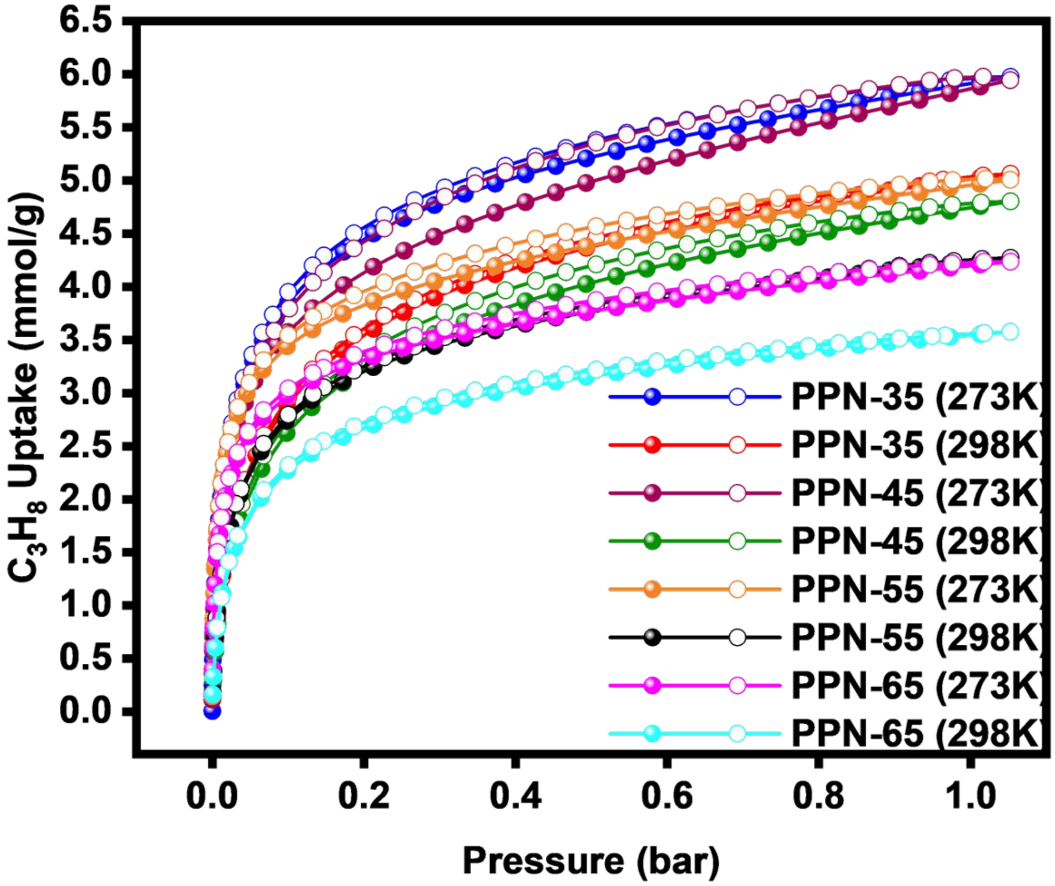

Figure S12. The C_3H_8 sorption isotherm of the four PPN sorbents (**PPN-35**, **PPN-45**, **PPN-55**, and **PPN-65**) at 273 K, 298 K, and 1 bar.

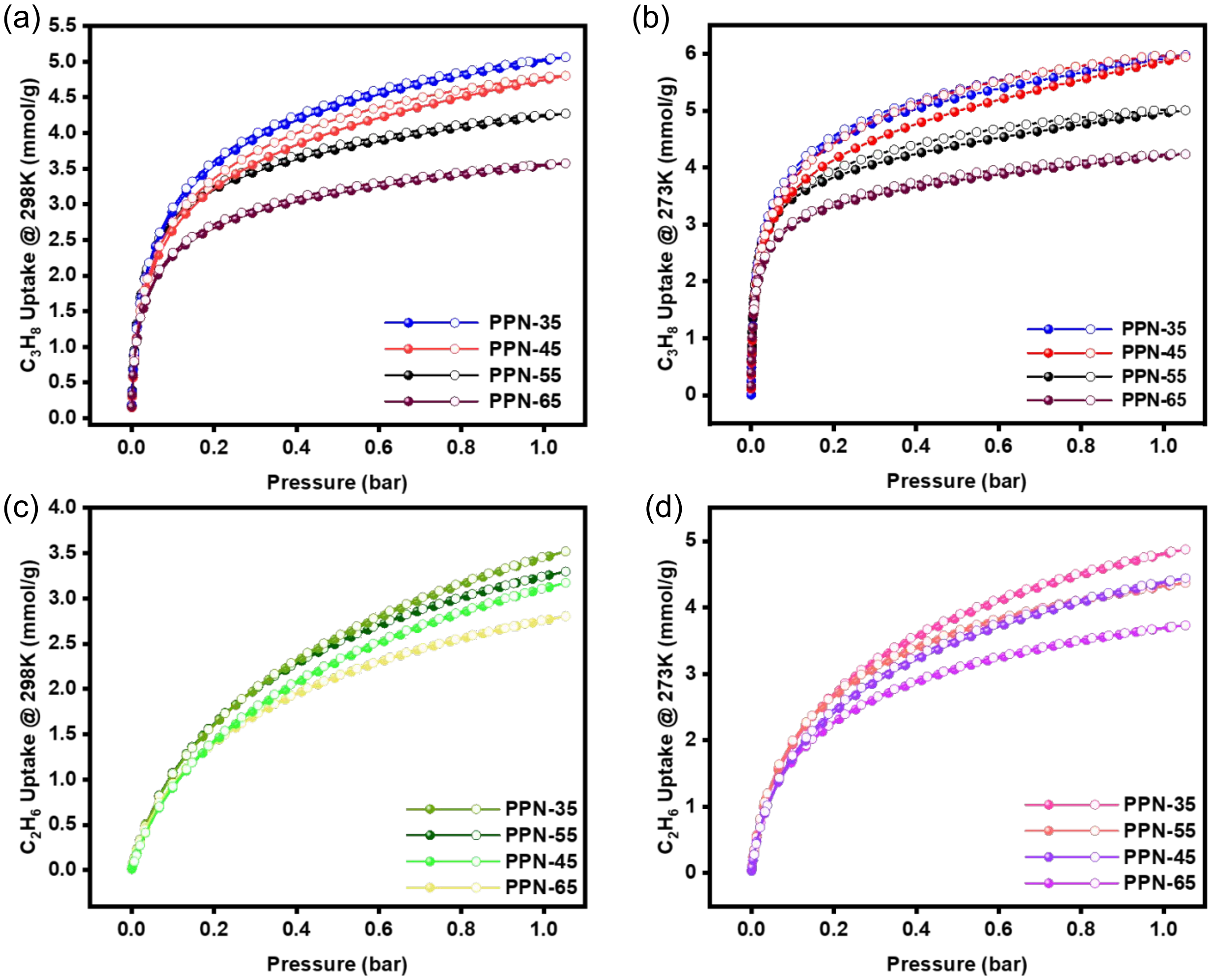

Figure S13. C_3H_8 sorption isotherms of **PPN-35** at (a) 298 K, (b) 273 K, and 1 bar. C_2H_6 sorption isotherm of **PPN-35** at (c) 298 K, (d) 273 K, and 1 bar.

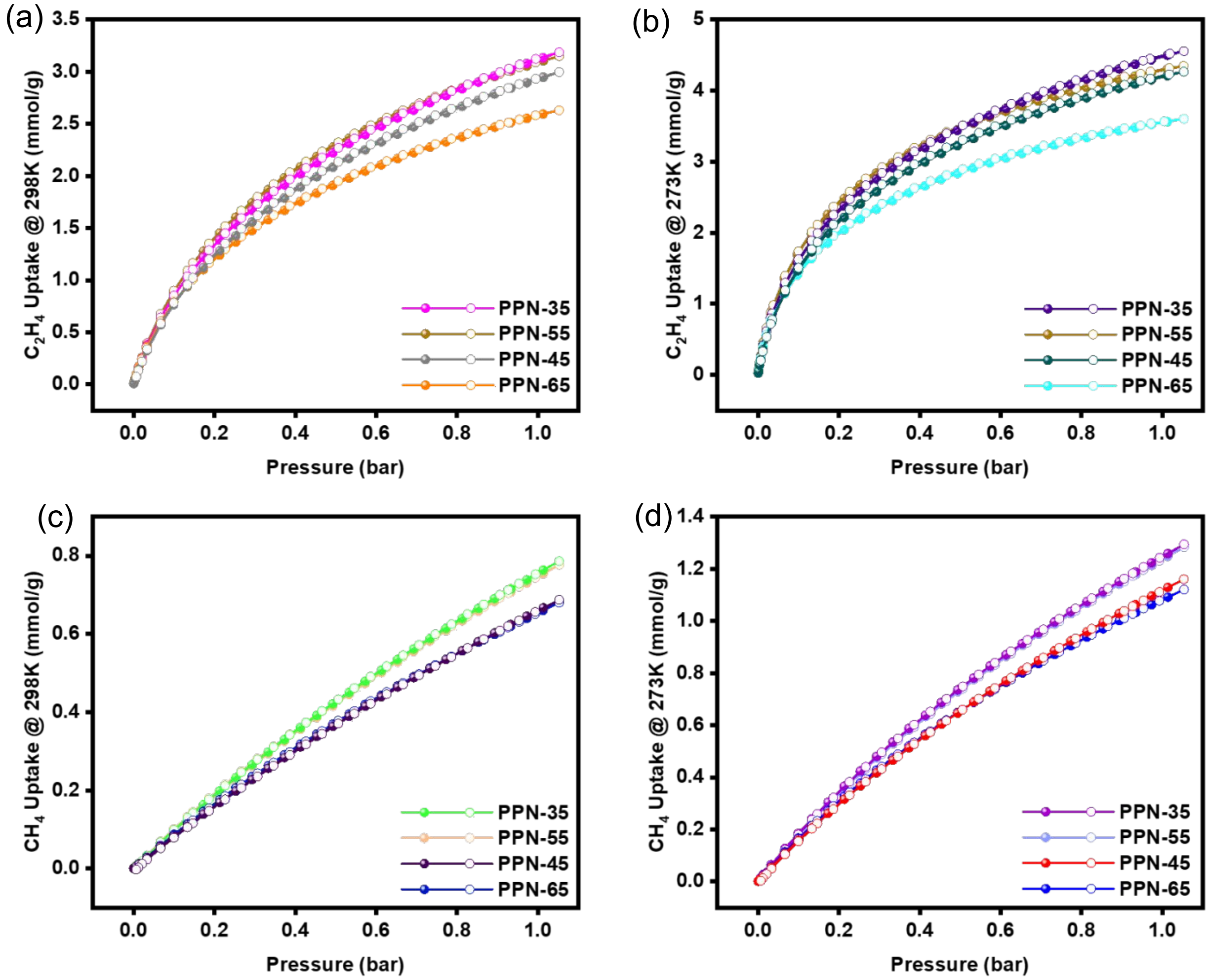

Figure S14. C_2H_4 sorption isotherms of **PPN-35** at (a) 298 K, (b) 273 K, and 1 bar. CH_4 sorption isotherm of **PPN-35** at (c) 298 K, (d) 273 K, and 1 bar.

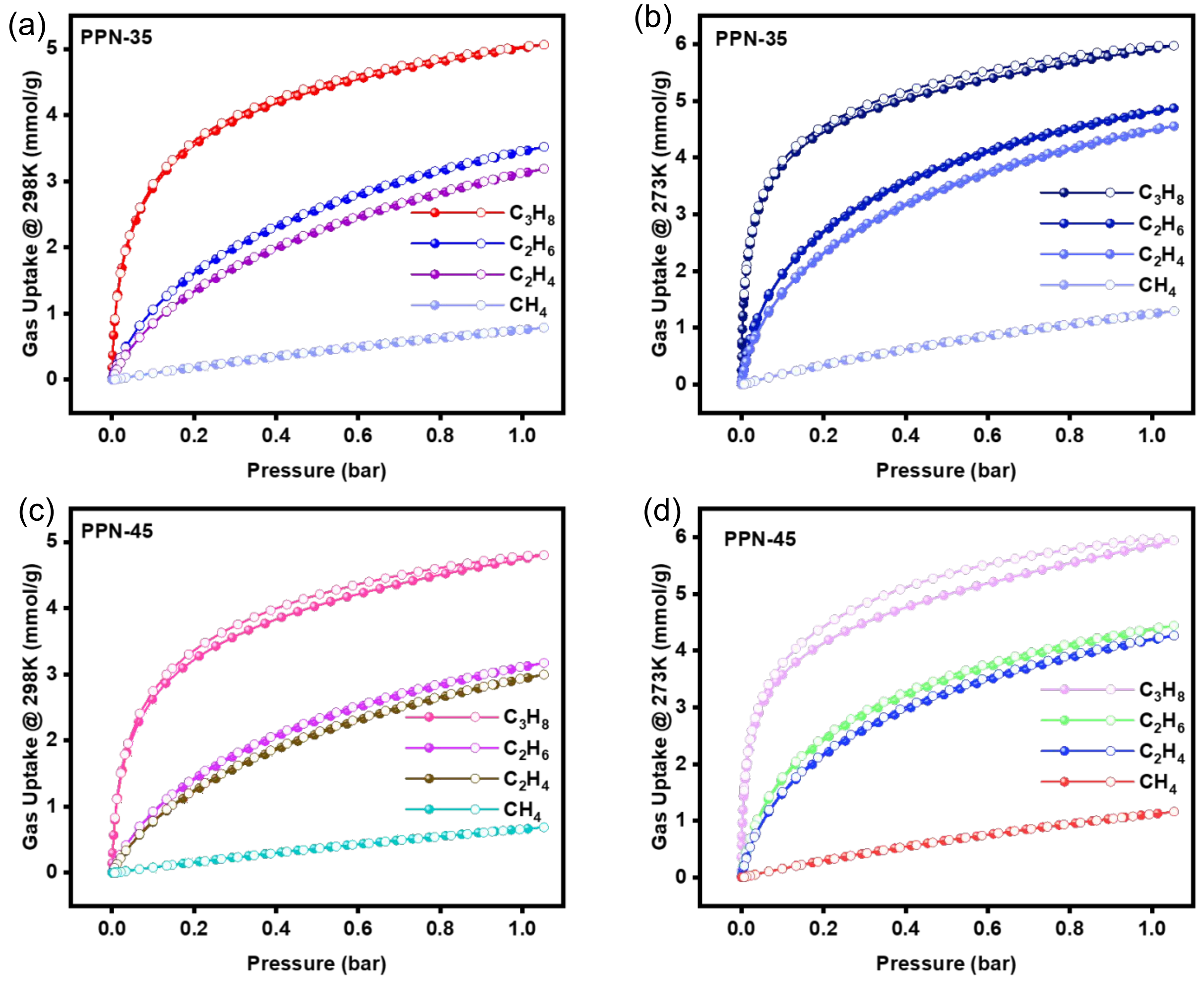

Figure S15. C_3H_8, C_2H_6, C_2H_4, and CH_4 sorption isotherms of **PPN-35** and **PPN-45** at 298 K (a, c) and 273 K (b, d) up to 1 bar.

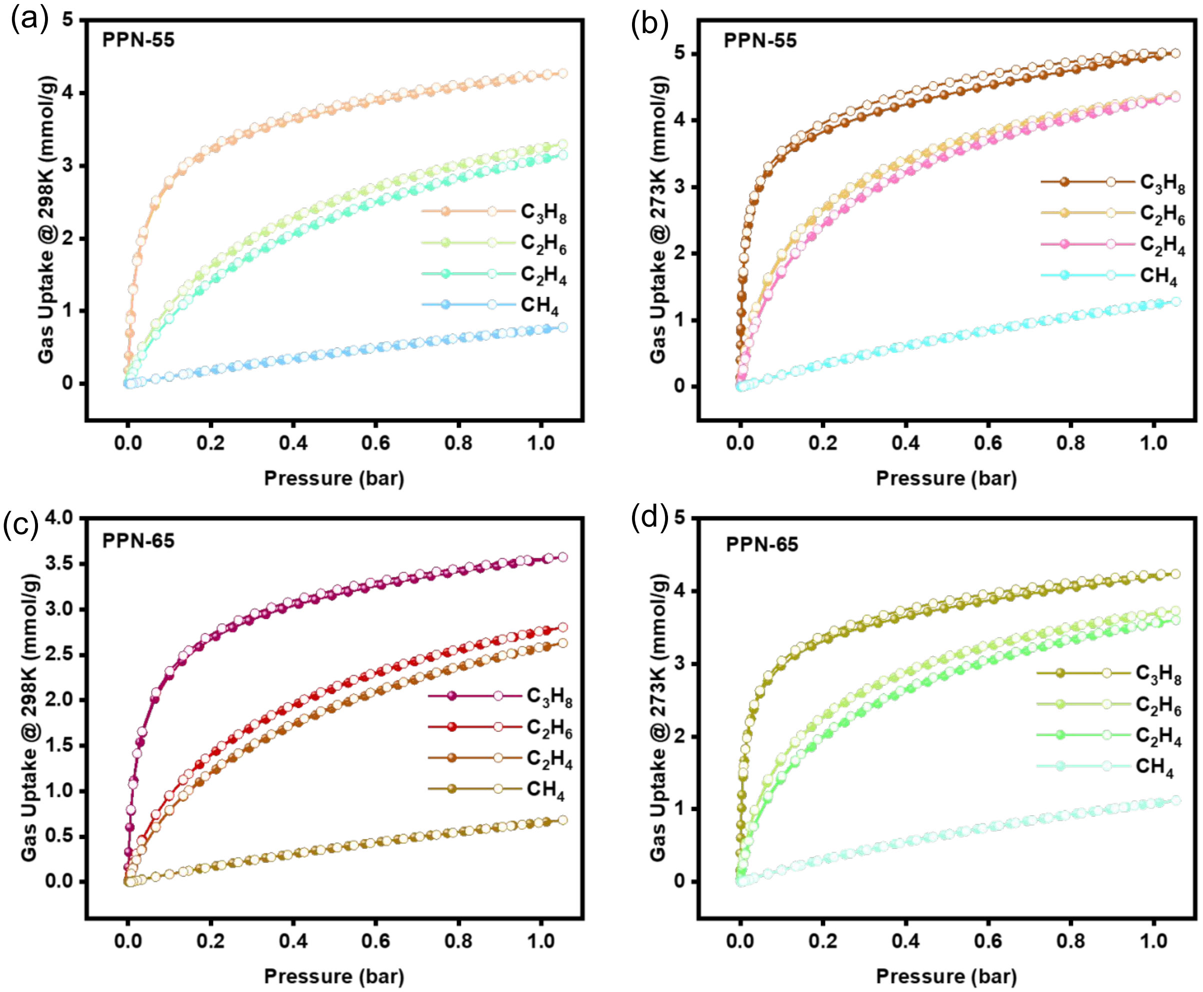

Figure S16. C_3H_8, C_2H_6, C_2H_4, and CH_4 sorption isotherms of **PPN-55** and **PPN-65** at 298 K (a, c) and 273 K (b, d) up to 1 bar.

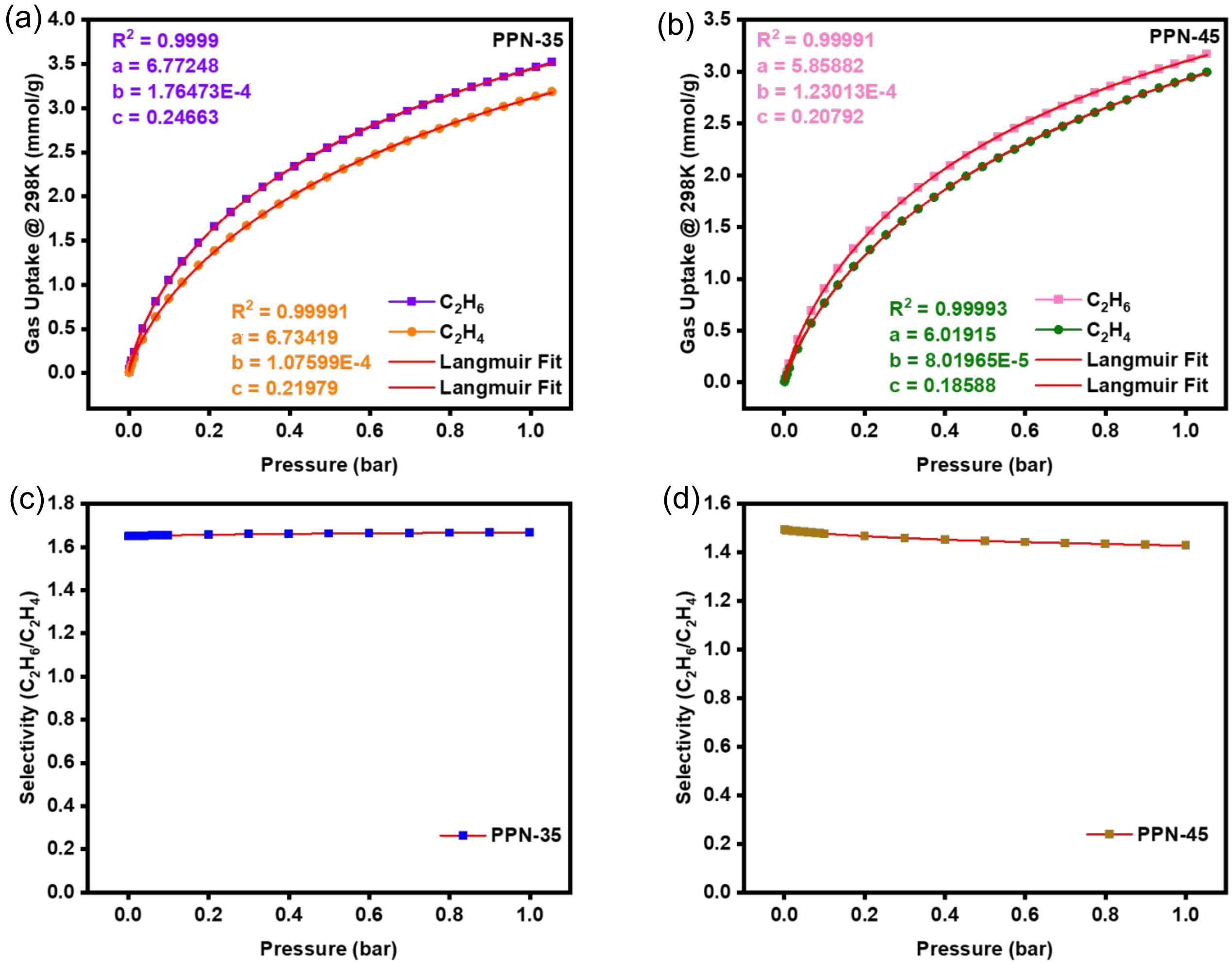

Figure S17. Extended Langmuir model fitting and IAST selectivity analysis for C_2H_6/C_2H_4 separation. (a) and (b) show the C_2H_6 and C_2H_4 adsorption isotherms at 298 K for **PPN-35** and **PPN-45**, respectively, fitted using the extended Langmuir equation. The corresponding fitting parameters and R^2 values are provided for each dataset. (c) and (d) display the IAST-predicted selectivity of C_2H_6 over C_2H_4 (50/50, v/v mixture) as a function of pressure at 298 K for **PPN-35** and **PPN-45**.

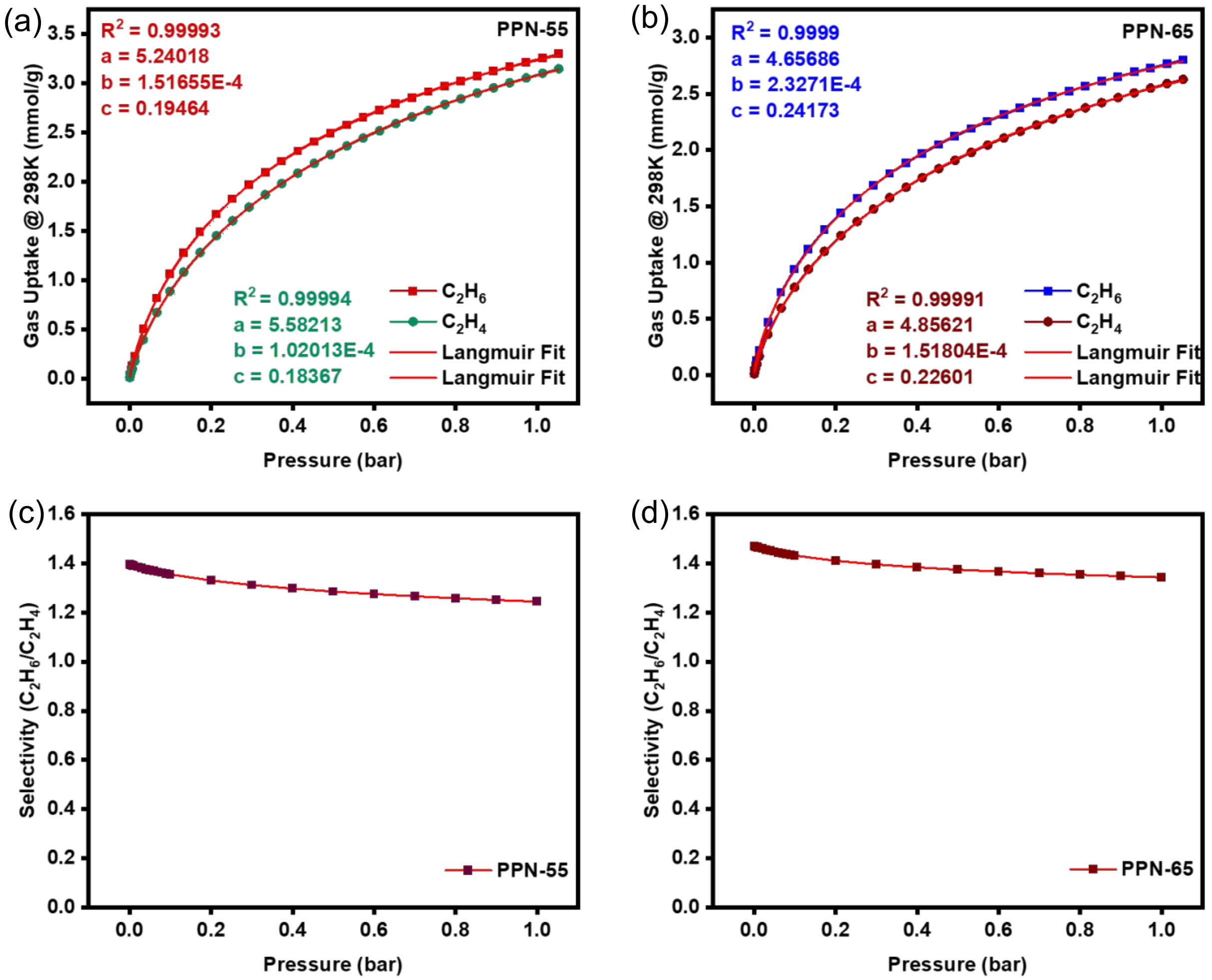

Figure S18. Extended Langmuir model fitting and IAST selectivity analysis for C_2H_6/C_2H_4 separation. (a) and (b) show the C_2H_6 and C_2H_4 adsorption isotherms at 298 K for **PPN-55** and **PPN-65**, respectively, fitted using the extended Langmuir equation. The corresponding fitting parameters and R^2 values are provided for each dataset. (c) and (d) display the IAST-predicted selectivity of C_2H_6 over C_2H_4 (50/50, v/v mixture) as a function of pressure at 298 K for **PPN-55** and **PPN-65**.

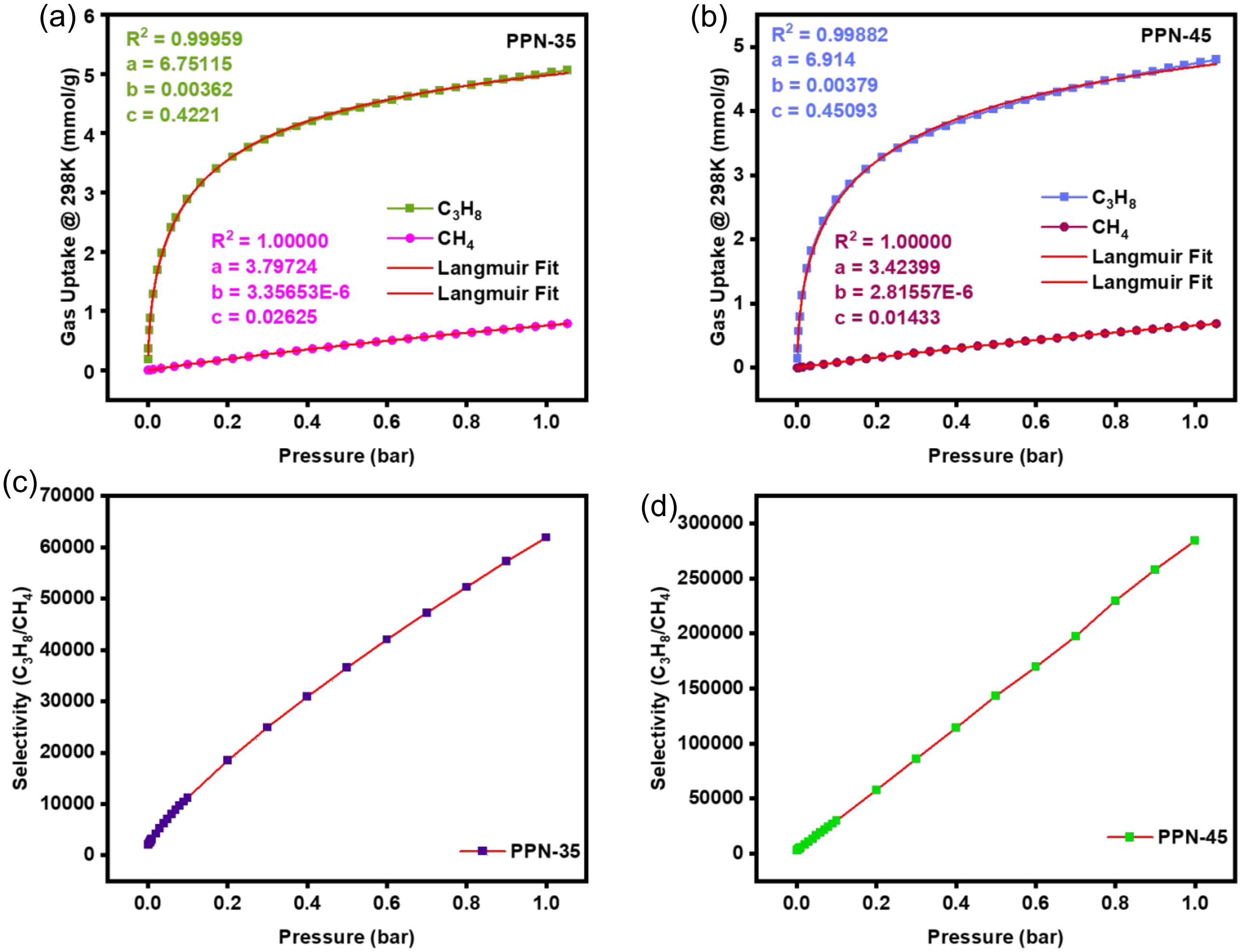

Figure S19. Extended Langmuir model fitting and IAST selectivity analysis for C_3H_8/CH_4 separation. (a) and (b) show the C_3H_8 and CH_4 adsorption isotherms at 298 K for **PPN-35** and **PPN-45**, respectively, fitted using the extended Langmuir equation. The corresponding fitting parameters and R^2 values are provided for each dataset. (c) and (d) display the IAST-predicted selectivity of C_3H_8 over CH_4 (50/50 v/v mixture) as a function of pressure at 298 K for **PPN-35** and **PPN-45**.

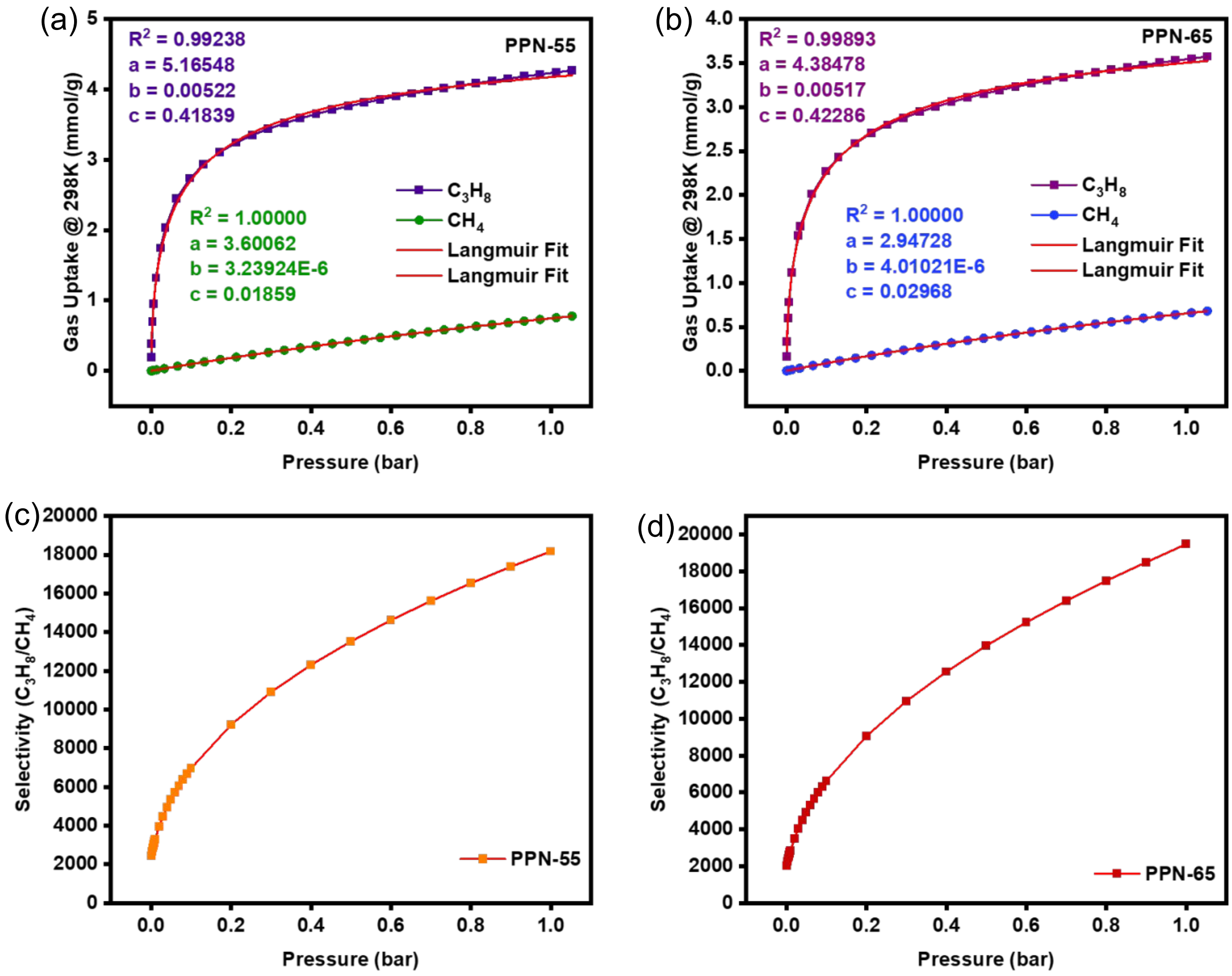

Figure S20. Extended Langmuir model fitting and IAST selectivity analysis for C_3H_8/CH_4 separation. (a) and (b) show the C_3H_8 and CH_4 adsorption isotherms at 298 K for **PPN-35** and **PPN-45**, respectively, fitted using the extended Langmuir equation. The corresponding fitting parameters and R^2 values are provided for each dataset. (c) and (d) display the IAST-predicted selectivity of C_3H_8 over CH_4 (50/50, v/v mixture) as a function of pressure at 298 K for **PPN-55** and **PPN-65**.

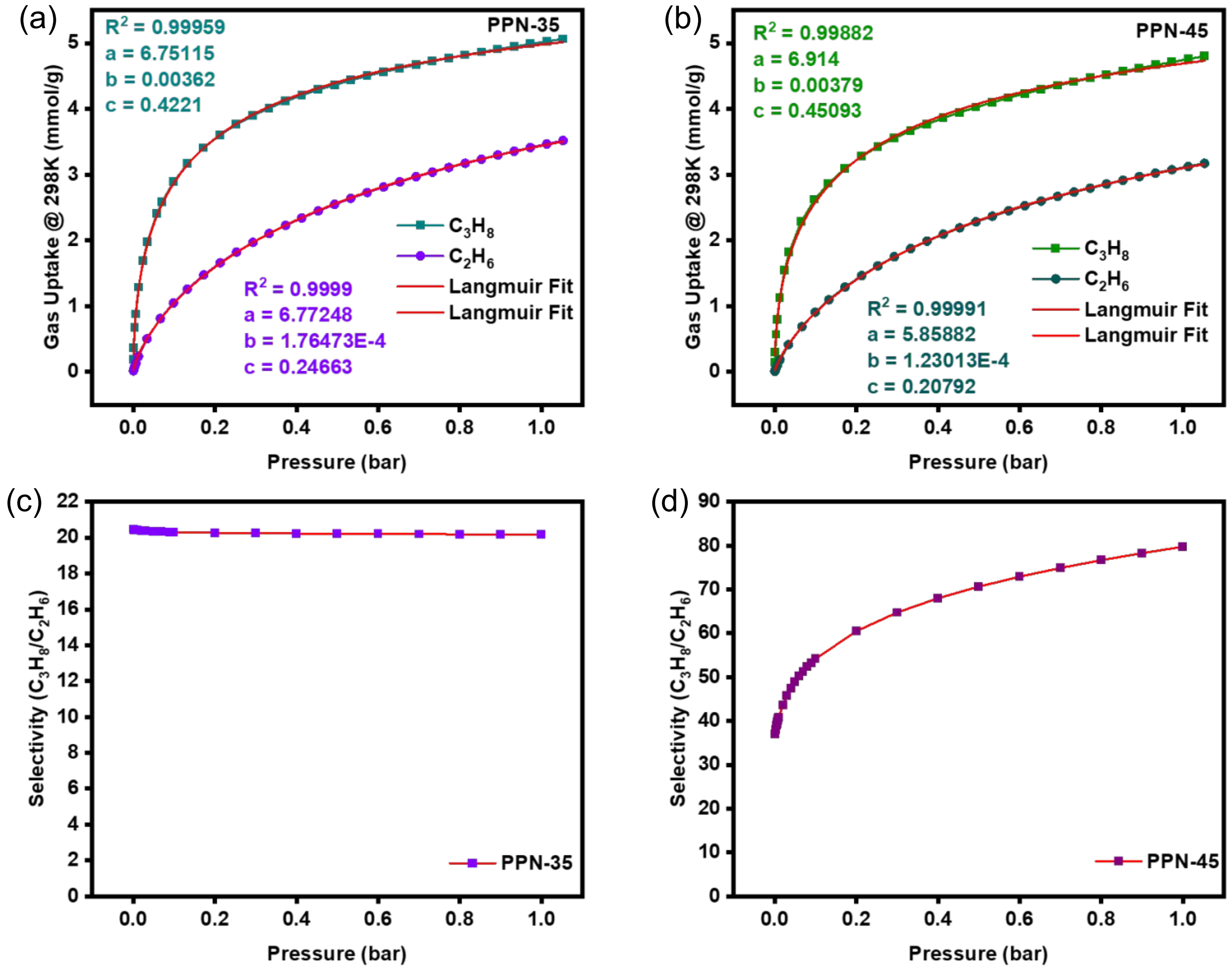

Figure S21. Extended Langmuir model fitting and IAST selectivity analysis for C_3H_8/C_2H_6 separation. (a) and (b) show the C_3H_8 and C_2H_6 adsorption isotherms at 298 K for **PPN-35** and **PPN-45**, respectively, fitted using the extended Langmuir equation. The corresponding fitting parameters and R^2 values are provided for each dataset. (c) and (d) display the IAST-predicted selectivity of C_3H_8 over C_2H_6 (50/50 v/v mixture) as a function of pressure at 298 K for **PPN-35** and **PPN-45**.

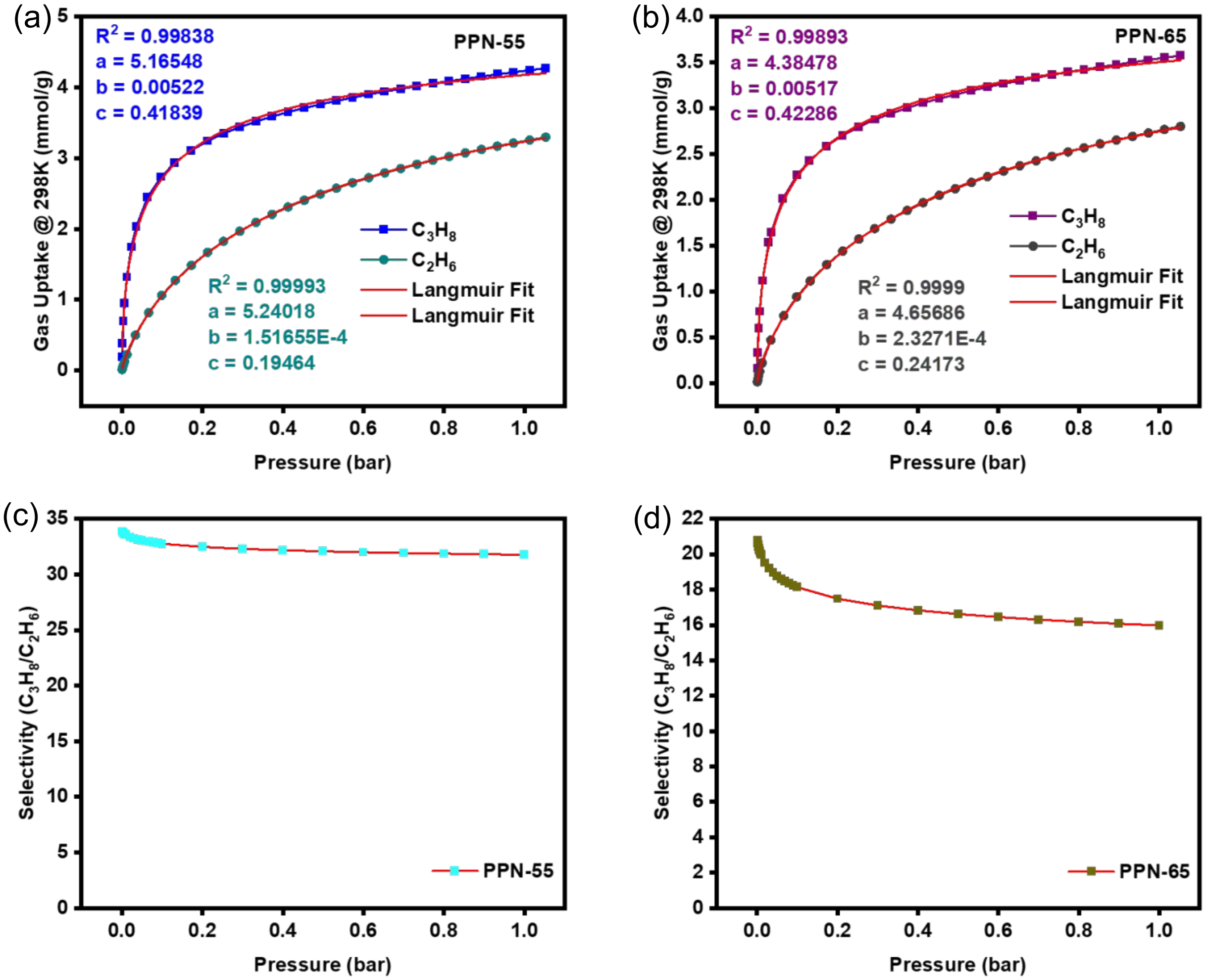

Figure S22. Extended Langmuir model fitting and IAST selectivity analysis for C_3H_8/C_2H_6 separation. (a) and (b) show the C_3H_8 and C_2H_6 adsorption isotherms at 298 K for **PPN-55** and **PPN-65**, respectively, fitted using the extended Langmuir equation. The corresponding fitting parameters and R^2 values are provided for each dataset. (c) and (d) display the IAST-predicted selectivity of C_3H_8 over C_2H_6 (50/50 v/v mixture) as a function of pressure at 298 K for **PPN-55** and **PPN-65**.

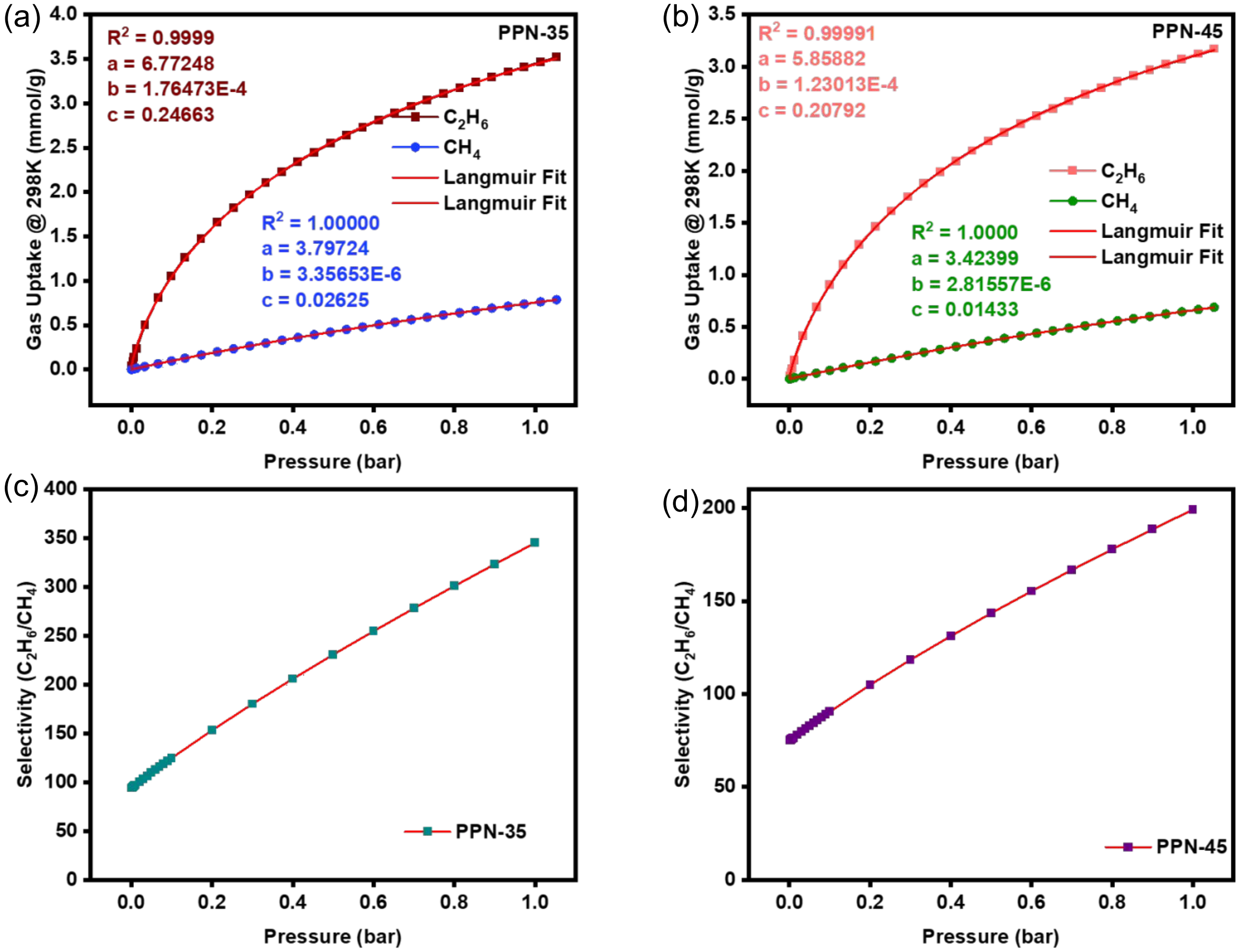

Figure S23. Extended Langmuir model fitting and IAST selectivity analysis for C_2H_6/CH_4 separation. (a) and (b) show the C_2H_6 and CH_4 adsorption isotherms at 298 K for **PPN-35** and **PPN-45**, respectively, fitted using the extended Langmuir equation. The corresponding fitting parameters and R^2 values are provided for each dataset. (c) and (d) display the IAST-predicted selectivity of C_2H_6 over CH_4 (50/50 v/v mixture) as a function of pressure at 298 K for **PPN-35** and **PPN-45**.

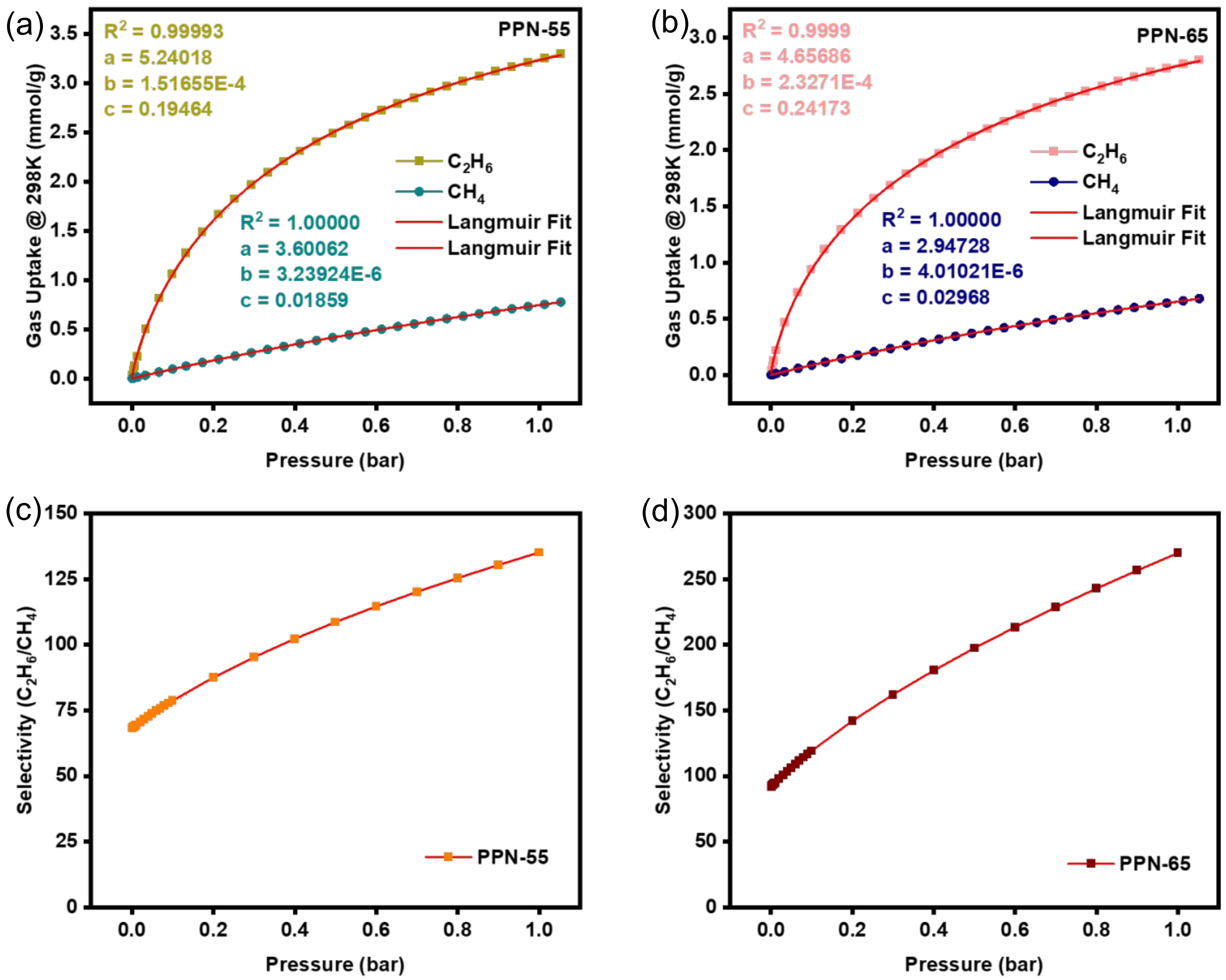

Figure S24. Extended Langmuir model fitting and IAST selectivity analysis for C_2H_6/CH_4 separation. (a) and (b) show the C_2H_6 and CH_4 adsorption isotherms at 298 K for **PPN-55** and **PPN-65**, respectively, fitted using the extended Langmuir equation. The corresponding fitting parameters and R^2 values are provided for each dataset. (c) and (d) display the IAST-predicted selectivity of C_2H_6 over CH_4 (50/50 v/v mixture) as a function of pressure at 298 K for **PPN-55** and **PPN-65**.

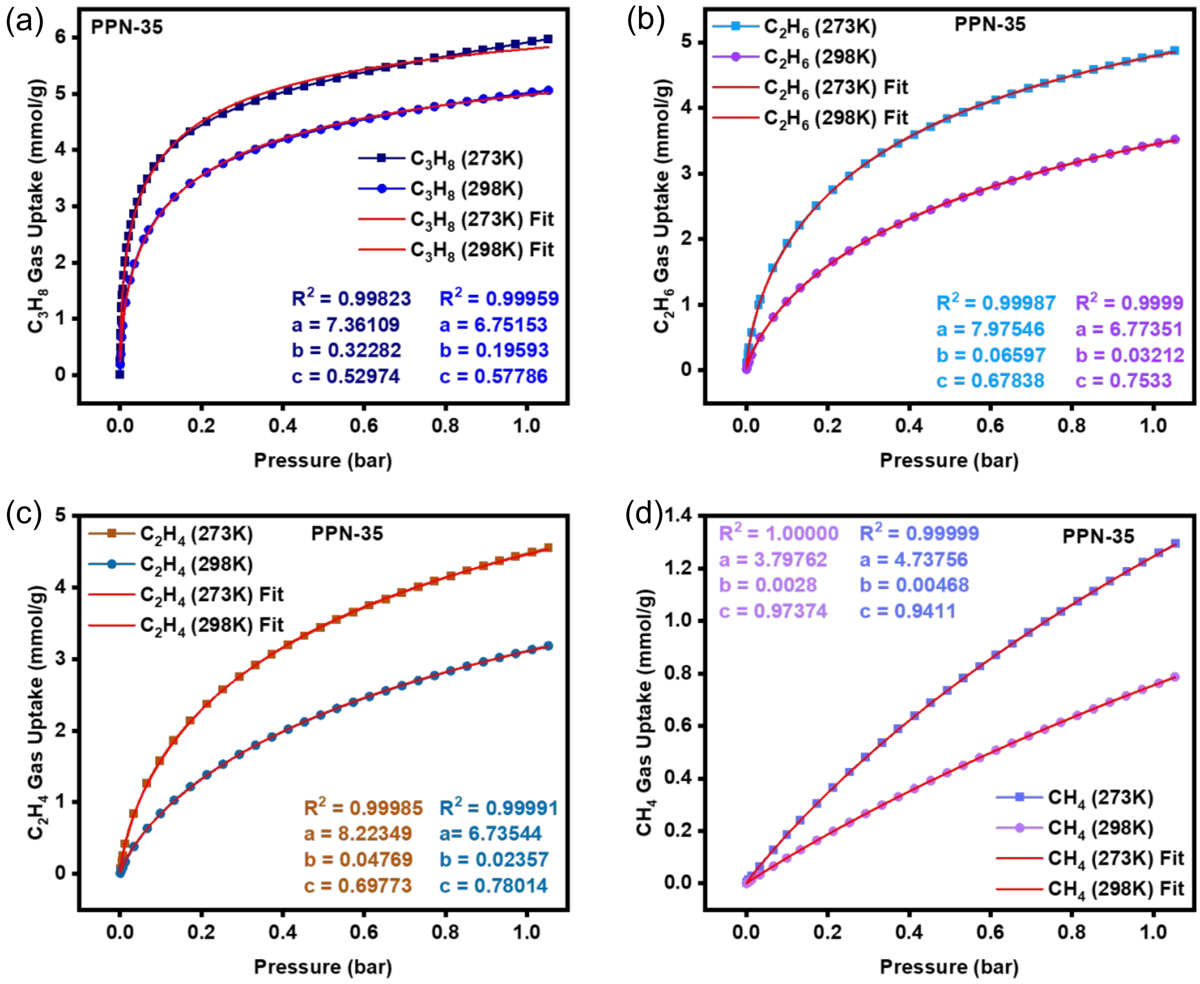

Figure S25. Langmuir–Freundlich isotherm fitting of gas adsorption data for **PPN-35** at 273 K and 298 K. (a) Propane (C_3H_8), (b) ethane (C_2H_6), (c) ethylene (C_2H_4), and (d) methane (CH_4) adsorption isotherms measured up to 1 bar. Experimental data are shown alongside Langmuir–Freundlich fits, with corresponding fitting parameters (a, b, c) and correlation coefficients (R^2) listed for each gas and temperature.

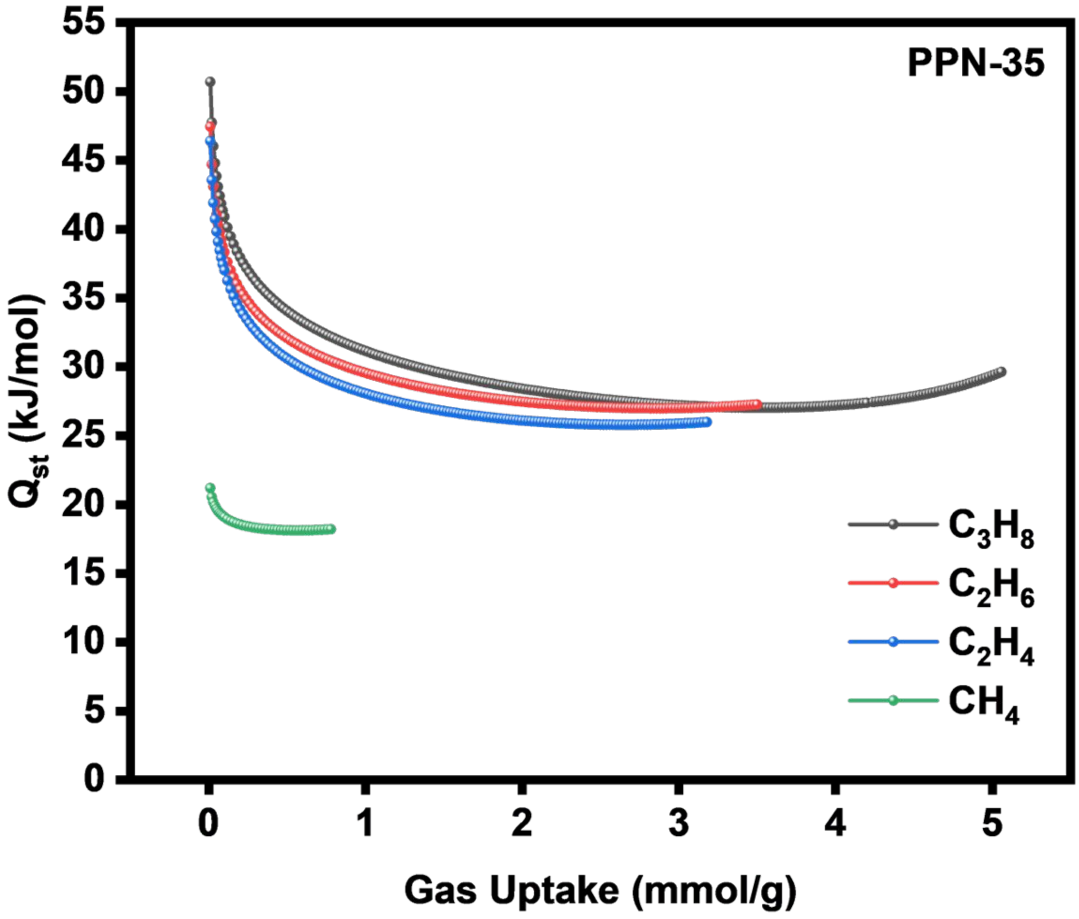

Figure S26. Isosteric heat of adsorption profiles for C_3H_8, C_2H_6, C_2H_4, and CH_4 on **PPN-35** as a function of gas uptake. The Q_{st} values were calculated using the Clausius–Clapeyron equation based on adsorption isotherms collected at 273 K and 298 K up to 1 bar.

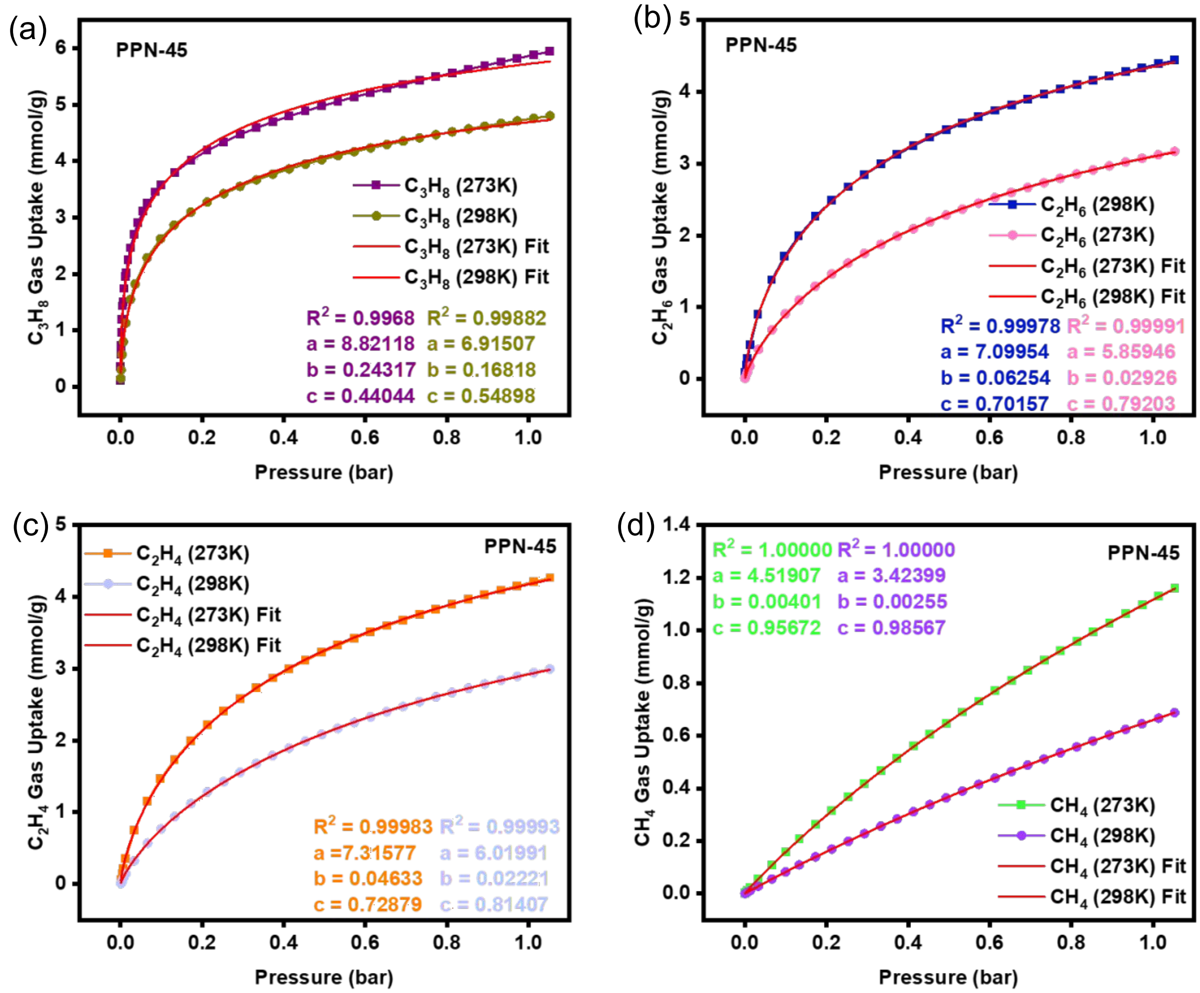

Figure S27. Langmuir–Freundlich isotherm fitting of gas adsorption data for **PPN-45** at 273 K and 298 K. (a) Propane (C_3H_8), (b) ethane (C_2H_6), (c) ethylene (C_2H_4), and (d) methane (CH_4) adsorption isotherms measured up to 1 bar. Experimental data are shown alongside Langmuir–Freundlich fits, with corresponding fitting parameters (a, b, c) and correlation coefficients (R^2) listed for each gas and temperature.

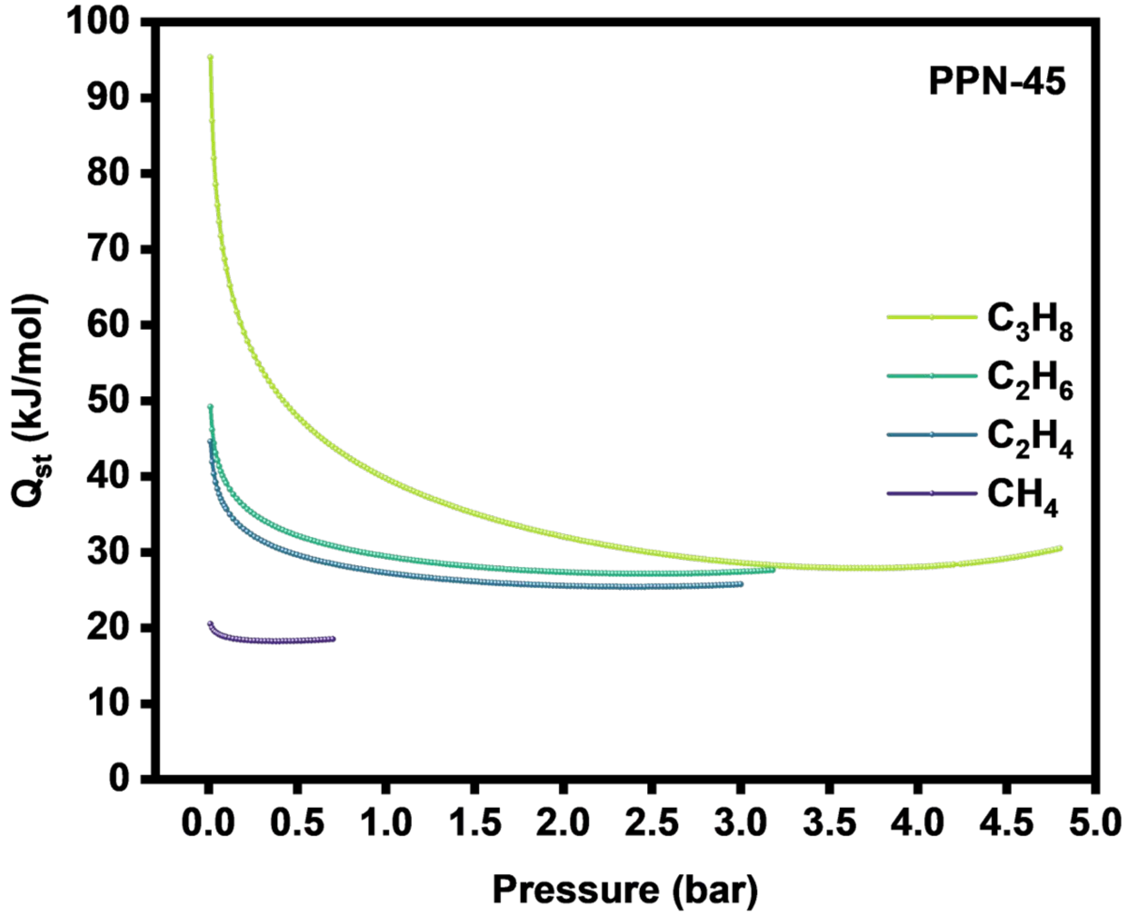

Figure S28. Isosteric heat of adsorption profiles for C_3H_8, C_2H_6, C_2H_4, and CH_4 on **PPN-45** as a function of gas uptake. The Q_{st} values were calculated using the Clausius–Clapeyron equation based on adsorption isotherms collected at 273 K and 298 K up to 1 bar.

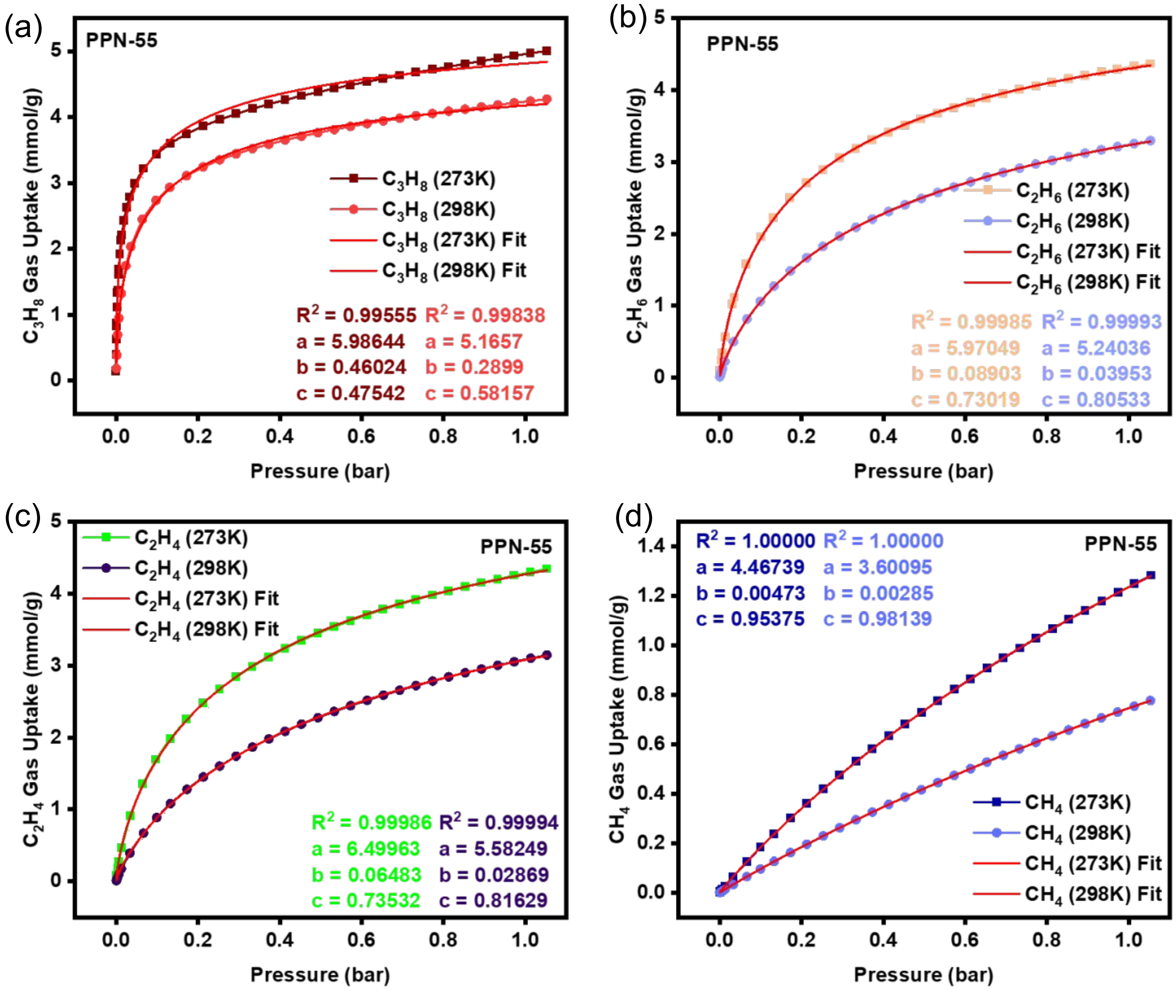

Figure S29. Langmuir–Freundlich isotherm fitting of gas adsorption data for **PPN-55** at 273 K and 298 K. (a) Propane (C_3H_8), (b) ethane (C_2H_6), (c) ethylene (C_2H_4), and (d) methane (CH_4) adsorption isotherms measured up to 1 bar. Experimental data are shown alongside Langmuir–Freundlich fits, with corresponding fitting parameters (a, b, c) and correlation coefficients (R^2) listed for each gas and temperature.

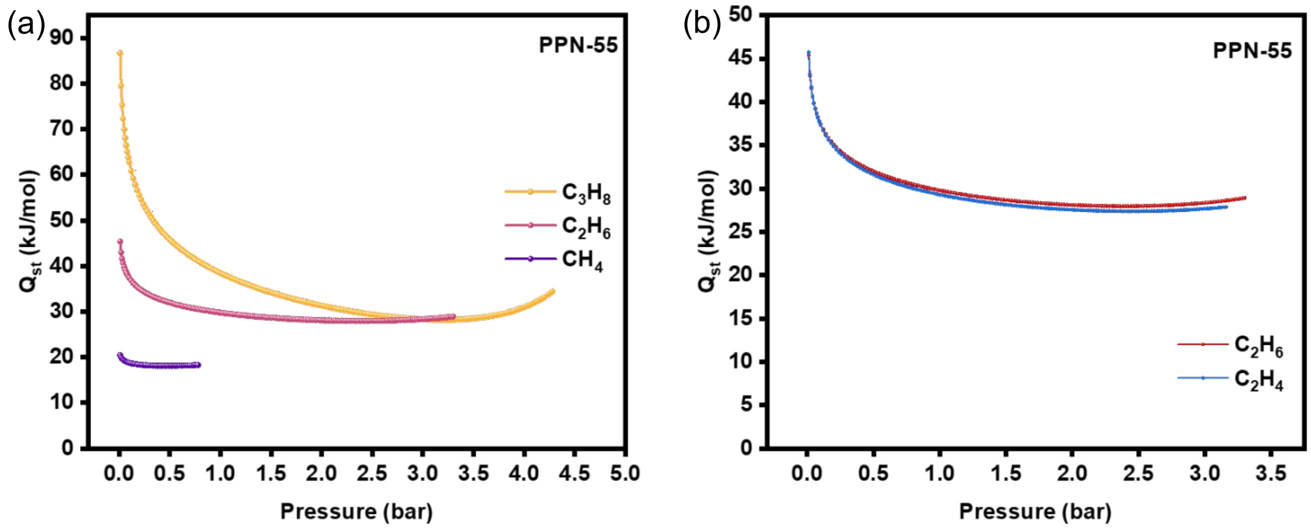

Figure S30. Isosteric heat of adsorption profiles for (a) C_3H_8, C_2H_6, CH_4, (b) C_2H_6, C_2H_4 on **PPN-55** as a function of gas uptake. The Q_{st} values were calculated using the Clausius–Clapeyron equation based on adsorption isotherms collected at 273 K and 298 K up to 1 bar.

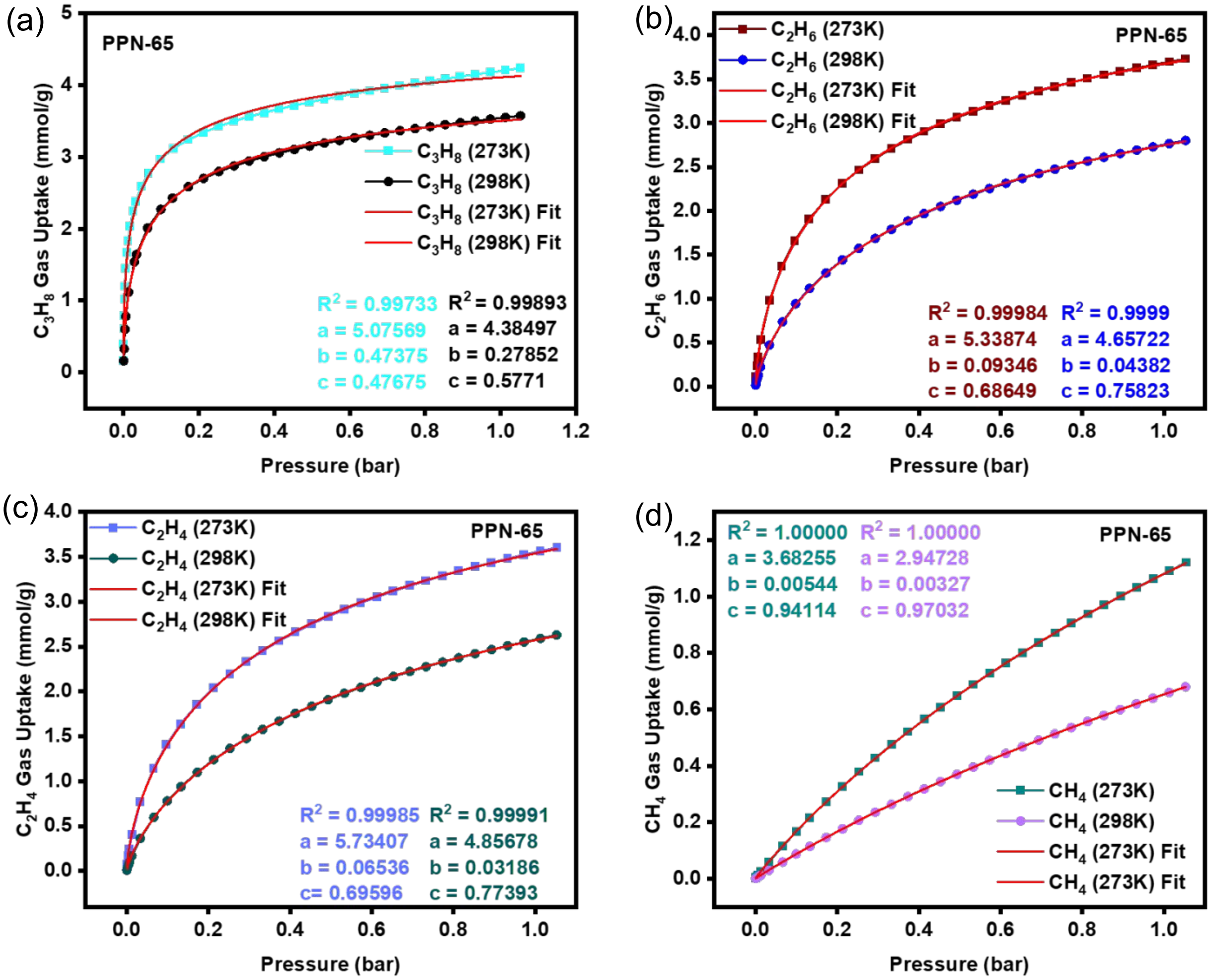

Figure S31. Langmuir–Freundlich isotherm fitting of gas adsorption data for **PPN-65** at 273 K and 298 K. (a) Propane (C_3H_8), (b) ethane (C_2H_6), (c) ethylene (C_2H_4), and (d) methane (CH_4) adsorption isotherms measured up to 1 bar. Experimental data are shown alongside Langmuir–Freundlich fits, with corresponding fitting parameters (a, b, c) and correlation coefficients (R^2) listed for each gas and temperature.

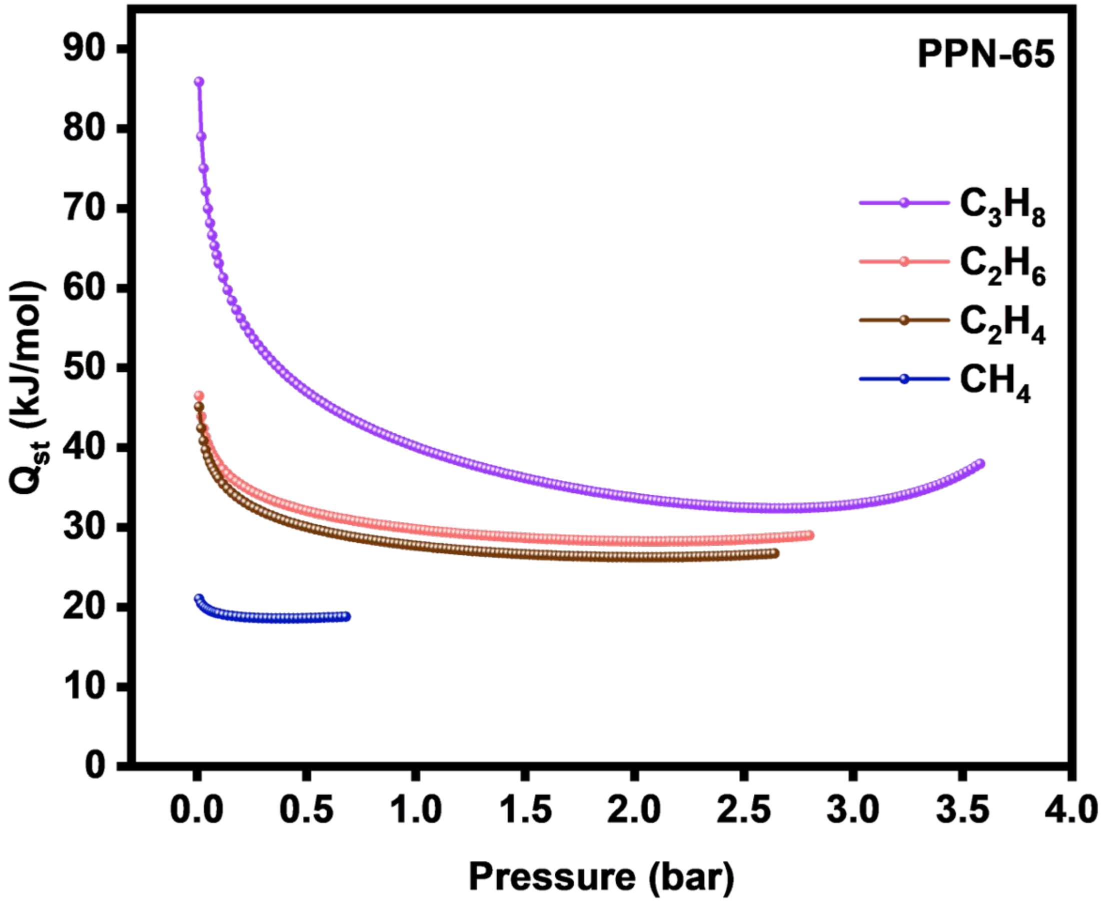

Figure S32. Isosteric heat of adsorption profiles for C_3H_8, C_2H_6, C_2H_4, and CH_4 on **PPN-65** as a function of gas uptake. The Q_{st} values were calculated using the Clausius–Clapeyron equation based on adsorption isotherms collected at 273 K and 298 K up to 1 bar.

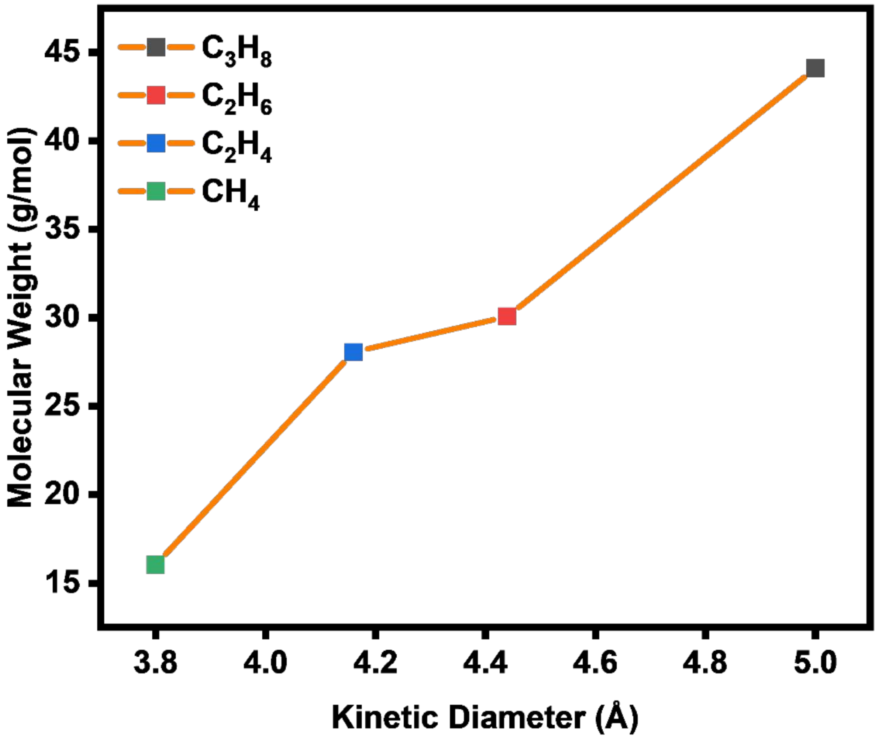

Figure S33. Correlation between molecular kinetic diameter and molecular weight for C_3H_8, C_2H_6, C_2H_4, and CH_4.

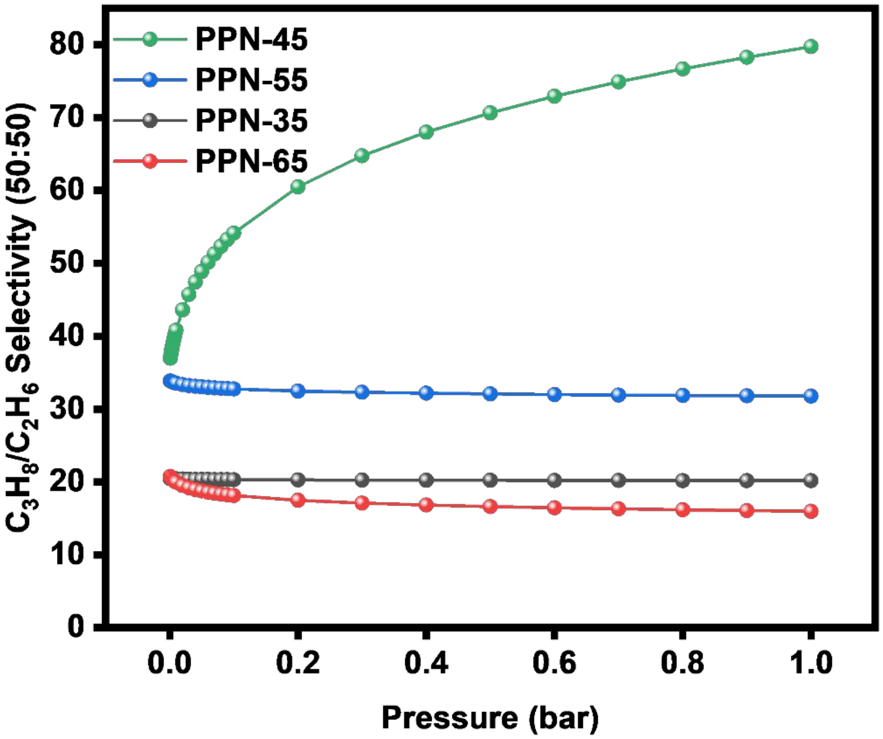

Figure S34. IAST-predicted selectivity of C_3H_8 over C_2H_6 for various PPN materials. The plot shows the selectivity values for **PPN-35**, **PPN-45**, **PPN-55**, and **PPN-65** as a function of pressure (0–1 bar) at 298 K for an equimolar (50/50, v/v) mixture of propane (C_3H_8) and ethane (C_2H_6).

Table S1. Gas adsorption parameters. Summary of the measured gas adsorption capacities (in mmol/g) for propane (C_3H_8), ethane (C_2H_6), ethylene (C_2H_4), and methane (CH_4) at 298 K and 1 bar on various porous polymer networks (**PPN-35**, **PPN-45**, **PPN-55**, and **PPN-65**).

Sorbents	Gas uptake at 298 K and 1 bar			
	C_3H_8	C_2H_6	C_2H_4	CH_4
**PPN-35**	5.06	3.52	3.18	0.79
**PPN-45**	4.80	3.17	2.99	0.69
**PPN-55**	4.27	3.30	3.14	0.78
**PPN-65**	3.57	2.80	2.62	0.68

Table S2. Physical properties of light hydrocarbon gases. The important physical properties of C_3H_8, C_2H_6, C_2H_4, and CH_4.

Gases	Polarization (10^{25} cm^3)	Boiling Points (K)	Kinetic Diameter (Å)	Molecular Weight (g/mol)	Quadruple Moment (10^{26} esu cm^2)
C_3H_8	62.9	231.2	5.0	44.09	-
C_2H_6	44.3-44.7	184.55	4.44	30.07	0.65
C_2H_4	42.52	169.42	4.16	28.05	1.50
CH_4	25.9	111.7	3.8	16.04	0

Table S3. IAST Selectivity and heat of adsorption parameters. IAST selectivity and isosteric heat of adsorption for light hydrocarbon gases on PPN materials. The table summarizes the IAST-predicted selectivity values for equimolar mixtures of C_2H_6/C_2H_4, C_2H_6/CH_4, C_3H_8/C_2H_6, and C_3H_8/CH_4 at 298 K and 1 bar for four sorbents: **PPN-35**, **PPN-45**, **PPN-55**, and **PPN-65**. Additionally, the corresponding isosteric heats of adsorption (Q_{st}, in kJ/mol) are reported for each gas on each material.

Sorbents	IAST Selectivity				Heat of Adsorption			
	C_2H_6/C_2H_4	C_2H_6/CH_4	C_3H_8/C_2H_6	C_3H_8/CH_4	C_3H_8	C_2H_6	C_2H_4	CH_4
**PPN-35**	1.67	345.33	20.17	61907.55	50.69	47.44	46.40	21.20
**PPN-45**	1.43	199.16	79.73	284358.59	95.37	49.22	44.62	20.55
**PPN-55**	1.24	135.21	31.77	18175.83	86.72	45.40	45.72	20.52
**PPN-65**	1.34	269.93	15.98	19484.73	85.86	46.48	45.12	21.02

Table S4. Heat of adsorption parameters. The heat of adsorption of the various light hydrocarbon gases on the individual sorbent at 298 K and 1 bar after full loading.

Sorbents	Heat of Adsorption @ 1 bar and full loading			
	C_3H_8	C_2H_6	C_2H_4	CH_4
**PPN-35**	29.63	27.27	25.99	18.20
**PPN-45**	30.51	27.65	25.77	18.53
**PPN-55**	34.40	28.91	27.88	18.31
**PPN-65**	37.96	28.98	29.71	18.80

Table S5. Summary of sorbents for C_2H_6/C_2H_4 selectivity. C_2H_6/C_2H_4 IAST selectivity comparison of best performing sorbents at 298 K and 1 bar.

Sorbent	C_2H_6 (mmol/g)	C_2H_4 (mmol/g)	C_2H_6/C_2H_4 Selectivity 50/50	References
**PPN-35**	**3.52**	**3.18**	**1.67**	**This work**
**PPN-45**	**3.17**	**2.99**	**1.43**	**This work**
**PPN-55**	**3.30**	**3.14**	**1.24**	**This work**
**PPN-65**	**2.80**	**2.62**	**1.34**	**This work**
$Fe_2(O_2)$(dobdc)	3.32	2.60	4.4	1
V-TBAPy	4.58	3.79	1.66	2
PCN-250	5.21	4.22	1.9	3
COF-1	2.46	1.92	1.9	4
MIL-160	4.40	4.12	1.37	5
MAF-49	2.70	1.70	1.7	6
3D-TPB-COF-H	3.25	3.23	1.4	7
3D-TPP-COF	4.93	4.70	1.8	
MIL-53-NDCA	4.24	3.12	1.53	8
$Cu(Qc)_2$	1.85	0.78	3.4	9
LIFM-63	3.0	2.1	1.56	10
PAN-AN	3.45	3.02	1.5	11
PAN-P	3.00	2.68	1.2	
Mn-PNM	2.75	2.02	1.38	12
CPOC-301	3.88	3.35	1.3	13
MIL-142A	3.8	2.9	1.5	14

Table S6. Summary of sorbents for C_3H_8/C_2H_6 selectivity. C_3H_8/C_2H_6 IAST selectivity comparison of best performing sorbents at 298 K and 1 bar.

Sorbents	C_3H_8/C_2H_6 Selectivity (50:50)	BET (m^2/g)	C_3H_8 (mmol/g)	C_2H_6 (mmol/g)	References
**PPN-35**	**20.17**	**1637**	**5.06**	**3.52**	**This work**
**PPN-45**	**79.73**	**1944**	**4.80**	**3.17**	**This work**
**PPN-55**	**31.77**	**1104**	**4.27**	**3.30**	**This work**
**PPN-65**	**15.98**	**1310**	**3.57**	**2.80**	**This work**
FJI-C1	6.00	2398.6	6.33	3.90	15
ZnP-CTF-400	9.00	1411	5.00	3.13	
ZnP-CTF-500	11.00	1848	7.19	4.02	16
ZnP-CTF-600	15.00	1331	4.59	2.41	
UPC-35	5.50	1087	4.97	1.82	17
NIIC-20-Et	29.0[a]	1023	5.60	2.38	
NIIC-20-Gl	25.2[a]	834	4.50	2.07	18
NIIC-20-Pr	28.0[a]	1130	5.50	2.41	

[a]at zero coverage

Table S7. Summary of sorbents for C_2H_6/CH_4 and C_3H_8/CH_4 selectivity. C_2H_6/CH_4 and C_3H_8/CH_4 IAST selectivity comparison of best performing sorbents at 298 K and 1 bar.

Sorbents	C_2H_6/CH_4 (50/50) Selectivity	C_3H_8/CH_4 (50/50) Selectivity	BET (m^2/g)	References
**PPN-35**	**345.33**	**61907.55**	**1637**	**This work**
**PPN-45**	**199.16**	**284358.59**	**1254**	**This work**
**PPN-55**	**135.21**	**18175.83**	**1104**	**This work**
**PPN-65**	**269.93**	**19484.73**	**1310**	**This work**
Co-MOF	83.8	715.6	697	[19]
ZUL-C1	22	73	417	[20]
ANPC-1-800	14.5	110.4	2836	[21]
ZUL-C2	91	632	430	[20]
JLU-Liu22	14.4	271.5	1487	[22]
NAC-700	65.7	501.9	2146.1	[23]
MIL-142A	13.7	1300	1424.65	[24]
FJI-C4	39.7	293.4	690	[25]
BSF-1	23	353	535	[26]
ANPC-2-700	13.5	162.5	2729	[21]
FJI-C1	22	471	1726.3	[15]
JLU-Liu40	21	845	1456	[27]
JLU-Liu18	13.1	108.2	1300	[28]

References

1. Li, L.; Lin, R.-B.; Krishna, R.; Li, H.; Xiang, S.; Wu, H.; Li, J.; Zhou, W.; Chen, B., Ethane/ethylene separation in a metal-organic framework with iron-peroxo sites. *Science* **2018,** *362* (6413), 443-446.
2. Li, M.; Mao, S.; Zhou, X.; Wang, H., One-step purification of ethylene from ethane/ethylene mixtures by a stable vanadium-based metal-organic framework. *Separation and Purification Technology* **2025,** *360*, 131049.
3. Chen, Y.; Qiao, Z.; Wu, H.; Lv, D.; Shi, R.; Xia, Q.; Zhou, J.; Li, Z., An ethane-trapping MOF PCN-250 for highly selective adsorption of ethane over ethylene. *Chemical Engineering Science* **2018,** *175*, 110-117.
4. He, C.; Wang, Y.; Chen, Y.; Wang, X.; Yang, J.; Li, L.; Li, J., Microregulation of Pore Channels in Covalent-Organic Frameworks Used for the Selective and Efficient Separation of Ethane. *ACS Applied Materials & Interfaces* **2020,** *12* (47), 52819-52825.
5. Cho, K. H.; Yoon, J. W.; Lee, J. H.; Kim, J. C.; Jo, D.; Park, J.; Lee, S. K.; Kwak, S. K.; Lee, U. H., Design of Pore Properties of an Al-Based Metal-Organic Framework for the Separation of an Ethane/Ethylene Gas Mixture via Ethane-Selective Adsorption. *ACS Appl Mater Interfaces* **2023,** *15* (25), 30975-30984.
6. Liao, P.-Q.; Zhang, W.-X.; Zhang, J.-P.; Chen, X.-M., Efficient purification of ethene by an ethane-trapping metal-organic framework. *Nature Communications* **2015,** *6* (1), 8697.
7. Xie, Y.; Wang, W.; Zhang, Z.; Li, J.; Gui, B.; Sun, J.; Yuan, D.; Wang, C., Fine-tuning the pore environment of ultramicroporous three-dimensional covalent organic frameworks for efficient one-step ethylene purification. *Nature Communications* **2024,** *15* (1), 3008.
8. Cho, K. H.; Yoon, J. W.; Lee, J. H.; Kim, J. C.; Kim, K.; Lee, U. H.; Choi, M.; Kwak, S. K.; Chang, J.-S., Pore control of Al-based MIL-53 isomorphs for the preferential capture of ethane in an ethane/ethylene mixture. *Journal of Materials Chemistry A* **2021,** *9* (25), 14593-14600.
9. Lin, R.-B.; Wu, H.; Li, L.; Tang, X.-L.; Li, Z.; Gao, J.; Cui, H.; Zhou, W.; Chen, B., Boosting Ethane/Ethylene Separation within Isoreticular Ultramicroporous Metal–Organic Frameworks. *Journal of the American Chemical Society* **2018,** *140* (40), 12940-12946.
10. Chen, C. X.; Wei, Z. W.; Pham, T.; Lan, P. C.; Zhang, L.; Forrest, K. A.; Chen, S.; Al-Enizi, A. M.; Nafady, A.; Su, C. Y.; Ma, S., Nanospace Engineering of Metal-Organic Frameworks through Dynamic Spacer Installation of Multifunctionalities for Efficient Separation of Ethane from Ethane/Ethylene Mixtures. *Angew Chem Int Ed Engl* **2021,** *60* (17), 9680-9685.
11. Wang, C.; Yan, J.; Ma, Z.; Wang, Z., Highly efficient separation of ethylene/ethane in microenvironment-modulated microporous polymers. *Separation and Purification Technology* **2022,** *287*, 120580.
12. Yang, L.; Wang, Y.; Chen, Y.; Yang, J.; Wang, X.; Li, L.; Li, J., Microporous metal-organic framework with specific functional sites for efficient removal of ethane from ethane/ethylene mixtures. *Chemical Engineering Journal* **2020,** *387*, 124137.
13. Su, K.; Wang, W.; Du, S.; Ji, C.; Yuan, D., Efficient ethylene purification by a robust ethane-trapping porous organic cage. *Nature Communications* **2021,** *12* (1), 3703.
14. Chen, Y.; Wu, H.; Lv, D.; Shi, R.; Chen, Y.; Xia, Q.; Li, Z., Highly Adsorptive Separation of Ethane/Ethylene by An Ethane-Selective MOF MIL-142A. *Industrial & Engineering Chemistry Research* **2018,** *57* (11), 4063-4069.
15. Huang, Y.; Lin, Z.; Fu, H.; Wang, F.; Shen, M.; Wang, X.; Cao, R., Porous Anionic Indium–Organic Framework with Enhanced Gas and Vapor Adsorption and Separation Ability. *ChemSusChem* **2014,** *7* (9), 2647-2653.

16. Ma, H.; Ren, H.; Meng, S.; Sun, F.; Zhu, G., Novel Porphyrinic Porous Organic Frameworks for High Performance Separation of Small Hydrocarbons. *Scientific Reports* **2013,** *3* (1), 2611.
17. Wang, Y.; Fan, W.; Wang, X.; Han, Y.; Zhang, L.; Liu, D.; Dai, F.; Sun, D., Solvent-induced framework-interpenetration isomers of Cu MOFs for efficient light hydrocarbon separation. *Inorganic Chemistry Frontiers* **2018,** *5* (10), 2408-2412.
18. Lysova, A. A.; Kovalenko, K. A.; Nizovtsev, A. S.; Dybtsev, D. N.; Fedin, V. P., Efficient separation of methane, ethane and propane on mesoporous metal-organic frameworks. *Chemical Engineering Journal* **2023,** *453*, 139642.
19. Wang, S.-M.; Xu, L.; Zhang, L.-P.; Li, Y.-T.; Wang, T.; Yang, Q.-Y., Rational Design of a π-Electron Rich Co-MOF Enabling Benchmark C2H6/CH4 Selectivity in Natural Gas Purification. *Advanced Functional Materials* **2025,** *n/a* (n/a), 2504251.
20. Zhou, J.; Ke, T.; Steinke, F.; Stock, N.; Zhang, Z.; Bao, Z.; He, X.; Ren, Q.; Yang, Q., Tunable Confined Aliphatic Pore Environment in Robust Metal–Organic Frameworks for Efficient Separation of Gases with a Similar Structure. *Journal of the American Chemical Society* **2022,** *144* (31), 14322-14329.
21. Zhang, P.; Wen, X.; Wang, L.; Zhong, Y.; Su, Y.; Zhang, Y.; Wang, J.; Yang, J.; Zeng, Z.; Deng, S., Algae-derived N-doped porous carbons with ultrahigh specific surface area for highly selective separation of light hydrocarbons. *Chemical Engineering Journal* **2020,** *381*, 122731.
22. Wang, D.; Liu, B.; Yao, S.; Wang, T.; Li, G.; Huo, Q.; Liu, Y., A polyhedral metal–organic framework based on the supermolecular building block strategy exhibiting high performance for carbon dioxide capture and separation of light hydrocarbons. *Chemical Communications* **2015,** *51* (83), 15287-15289.
23. Wang, J.; Krishna, R.; Yang, T.; Deng, S., Nitrogen-rich microporous carbons for highly selective separation of light hydrocarbons. *Journal of Materials Chemistry A* **2016,** *4* (36), 13957-13966.
24. Yuan, Y.; Wu, H.; Xu, Y.; Lv, D.; Tu, S.; Wu, Y.; Li, Z.; Xia, Q., Selective extraction of methane from C1/C2/C3 on moisture-resistant MIL-142A with interpenetrated networks. *Chemical Engineering Journal* **2020,** *395*, 125057.
25. Li, L.; Wang, X.; Liang, J.; Huang, Y.; Li, H.; Lin, Z.; Cao, R., Water-Stable Anionic Metal-Organic Framework for Highly Selective Separation of Methane from Natural Gas and Pyrolysis Gas. *ACS Appl Mater Interfaces* **2016,** *8* (15), 9777-81.
26. Zhang, Y.; Yang, L.; Wang, L.; Duttwyler, S.; Xing, H., A microporous metal-organic framework supramolecularly assembled from a CuII dodecaborate cluster complex for selective gas separation. *Angewandte Chemie* **2019,** *131* (24), 8229-8234.
27. Sun, Q.; Yao, S.; Liu, B.; Liu, X.; Li, G.; Liu, X.; Liu, Y., A novel polyhedron-based metal–organic framework with high performance for gas uptake and light hydrocarbon separation. *Dalton Transactions* **2018,** *47* (14), 5005-5010.
28. Yao, S.; Wang, D.; Cao, Y.; Li, G.; Huo, Q.; Liu, Y., Two stable 3D porous metal–organic frameworks with high performance for gas adsorption and separation. *Journal of Materials Chemistry A* **2015,** *3* (32), 16627-16632.
